# G-protein-coupled receptor participates in 20-hydroxyecdysone signaling on the plasma membrane

**DOI:** 10.1186/1478-811X-12-9

**Published:** 2014-02-10

**Authors:** Mei-Juan Cai, Du-Juan Dong, Yu Wang, Peng-Cheng Liu, Wen Liu, Jin-Xing Wang, Xiao-Fan Zhao

**Affiliations:** 1 The Key Laboratory of Plant Cell Engineering and Germplasm Innovation, Ministry of Education, Shandong Provincial Key Laboratory of Animal Cells and Developmental Biology, School of Life Sciences, Shandong University, Jinan 250100, Shandong, China

**Keywords:** Steroid hormones, G-protein-coupled receptors, Protein phosphorylation, Calcium influx, Signal transduction

## Abstract

**Background:**

Animal steroid hormones are conventionally known to initiate signaling via a genomic pathway by binding to the nuclear receptors. The mechanism by which 20E initiates signaling via a nongenomic pathway is unclear.

**Results:**

We illustrate that 20E triggered the nongenomic pathway through a plasma membrane G-protein-coupled receptor (named ErGPCR) in the lepidopteran insect *Helicoverpa armigera*. The transcript of *ErGPCR* was increased at the larval molting stage and metamorphic molting stage by 20E regulation. Knockdown of *ErGPCR* via RNA interference *in vivo* blocked larval–pupal transition and suppressed 20E-induced gene expression. ErGPCR overexpression in the *H. armigera* epidermal cell line increased the 20E-induced gene expression. Through ErGPCR, 20E modulated Calponin nuclear translocation and phosphorylation, and induced a rapid increase in cytosolic Ca^2+^ levels. The inhibitors of T-type voltage-gated calcium channels and canonical transient receptor potential calcium channels repressed the 20E-induced Ca^2+^ increase. Truncation of the N-terminal extracellular region of ErGPCR inhibited its localization on the plasma membrane and 20E-induced gene expression. ErGPCR was not detected to bind with the steroid hormone analog [^3^H]Pon A.

**Conclusion:**

These results suggest that ErGPCR participates in 20E signaling on the plasma membrane.

## Background

Animal steroid hormones are lipid-soluble molecules conventionally known to initiate signaling via a genomic pathway. Steroid hormones enter the nucleus by freely diffusing through cell membranes to combine with intracellular nuclear receptors for gene transactivation. Nuclear receptors act by forming homodimers or heterodimers. For example, the glucocorticoid receptor [1] and estrogen receptor [2] form a homodimer, and insect ecdysone receptor (EcR) forms a heterodimer with an ultraspiracle protein (USP), the ortholog of retinoid X receptor in vertebrates [3]. The heat shock proteins Hsp90 and Hsp70 interact with the nuclear receptors to facilitate their DNA binding activity in fruit flies [4] and mammals [5]. Hsp90 [6] and Hsc70 [7] have been found to be involved in insect steroid hormone signaling by differential interaction with the nuclear receptors. However, plant steroid hormones, such as brassinosteroids, employ a nongenomic pathway to initiate signaling by combining with plasma membrane receptor kinases for gene transactivation [8]. The brassinosteroids exhibit structural similarity to the steroid hormones of vertebrates and insects [9]. These studies suggest the existence of similar pathways in plants and animals, which should be further studied.

Previous studies indicated that animal steroid hormones can trigger nongenomic actions through the cytoplasmic membrane [10]. For example, estrogen activates phosphoinositide 3 kinase to recruit protein kinase B to the membrane in mammals [11]. A G-protein-coupled seven transmembrane receptor (GPR30) can act as the membrane receptor for estrogen [12], and it has been renamed G-protein-coupled estrogen receptor 1 (GPER) [13]. However, GPR30 is located in the endoplasmic reticulum [14] and may be translocated to the plasma membrane [15]. New studies have suggested that GPER is ubiquitinated at the cell surface, and constitutively internalized in an arrestin-independent manner. Moreover, GPER does not recycle to the plasma membrane [16]. GPER acts as a stand-alone membrane receptor of pregenomic action independent on the estrogen nuclear receptor [17]. GPER also functions in the nervous system, and may be a pharmaceutical target [18].

In insects, the ATP-binding cassette transporter E23 can act as a general negative regulator of ecdysteroid signaling by transporting 20E outside of the cell [19]. 20E promotes neuroblast proliferation during metamorphosis partly by suppressing nitric oxide production in <15 min without protein synthesis or transcription [20], and the cell membrane receptor of 20E is assumed to be a leucine-rich repeat receptor kinase [21]. The plasma membrane of the anterior silk gland of *Bombyx mori* binds [^3^H] ponasterone A ([^3^H]Pon A), suggesting that the anterior silk gland may express an unknown membrane 20E receptor [22]. 20E induces intracellular Ca^2+^ release into the cytoplasm via an unknown G-protein-coupled receptor (GPCR) pathway in the anterior silk gland of silkworms [23]. The *Drosophila* dopamine receptor DmDopEcR binds [^3^H]Pon A, and is considered as a 20E membrane receptor [24]. Ecdysteroids trigger rapid Ca^2+^ increase, including intracellular Ca^2+^ release, and extracellular Ca^2+^ influx through GPCR in mouse skeletal muscle cells [25]. In our previous study, we demonstrated that 20E regulates the rapid nuclear translocation and phosphorylation of Calponin for gene expression in *Helicoverpa armigera*[26]. These findings suggest that 20E has membrane receptors and a nongenomic signaling pathway.

In this study, we reported an ecdysone-responsible GPCR (ErGPCR) participates in 20E signaling on the plasma membrane. The knockdown of ErGPCR disrupted several biological processes, including the larval–pupal metamorphosis, expression of 20E-induced genes, subcellular translocation and phosphorylation of Calponin, and 20E-induced cytosolic Ca^2+^ increase.

## Results

### *ErGPCR* is involved in 20E-regulated gene expression

**Figure 1:**
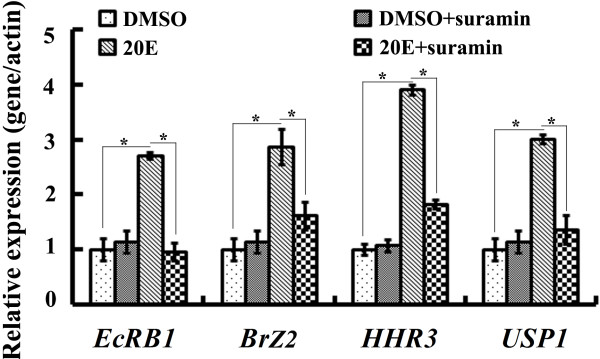
**Involvement of GPCRs in the 20E pathway in HaEpi cells as determined by quantitative real-time reverse transcription polymerase chain reaction (qRT-PCR) analysis.** DMSO treatment was used as the solvent control for 20E. DMSO plus suramin 50 μM treatment for 1 h was used to determine the toxic effects of suramin on the cells. The HaEpi cells were pretreated with 50 μM suramin for 1 h and then exposed to 1 μM 20E for another 6 h. The results are based on the ΔΔCT calculation by normalization of the *β-actin* gene. Error bars represent the standard deviation of three independent replicates. Asterisks indicate significant differences (Student’s *t* test, *p < 0.05).

It has been known that 20E regulates the gene expression of the nuclear receptor *EcRB1* and transcription factors *Br*, *USP1*, *E75B*, and *HR3*[27]. Suramin disrupts GPCR binding with the G protein by blocking the association of G protein α and βγ subunits [28]. Suramin is widely used to study GPCR- and G-protein-initiated cell signaling, including the 20E-induced GPCR pathway in the anterior silk gland of silkworms [23], cytosolic Ca^2+^ increase, and protein kinase C activation [29]. Thus, the involvement of GPCRs in 20E-induced gene expression was analyzed using the GPCR inhibitor suramin in a lepidopteran *H. armigera* epidermal cell line (HaEpi cell line, established in our laboratory) [30]. 20E significantly promoted the expression of *EcRB1*, *BrZ2*, *HHR3*, and *USP1* compared with the DMSO solvent control. However, the 20E-induced transcript increase was repressed by the addition of suramin (Figure 1). These results suggest that GPCRs are probably involved in 20E-regulated mRNA levels.

We identified six GPCR candidates from the expressed sequence tags (EST) of the cDNA library of the HaEpi cell line using BLASTX assay (http://www.ncbi.nlm.nih.gov/) (Additional file 1: Figure S1, Table S1). The mRNA levels of six GPCR candidates were upregulated by 20E induction, and two non-GPCR ESTs were unaffected. Knockdown of No. 16666 and ErGPCR in the HaEpi cells using RNA interference (RNAi) decreased *EcRB1*, *BrZ2*, *HHR3*, and *USP1* transcript levels in 20E induction. The knockdown of the other four GPCR candidates affected one to three 20E-induced gene transcripts (Additional file 1: Figure S2). These results suggest the involvement of GPCRs in 20E-induced gene expression.

*ErGPCR* was further studied regarding its expression profile during development. The deduced amino acid sequence of ErGPCR contains a signal peptide at the N-terminus and seven transmembrane domains (Additional file 1: Figure S3). ErGPCR belongs to methuselah-like proteins in the class B secretin GPCR family based on NCBI Blast analysis (http://blast.ncbi.nlm.nih.gov/Blast.cgi). ErGPCR has 57% identity with *Spodoptera frugiperda* GPCR, 32% with *Tribolium castaneum* GPCR, and 30% with *Drosophila melanogaster* GPCR (Additional file 1: Figure S4). However, *D. melanogaster* DmDopEcR, *Homo sapiens* GPR30, and *H. sapiens* beta-2 adrenergic receptor (AR) are not found by BLASTX analysis. This finding suggests that ErGPCR is less similar to DmDopEcR, GPR30, and AR. Phylogenetic analysis indicated that ErGPCR does not cluster with DmDopEcR, GPR30, and AR. These results illustrate that these GPCRs belong to different GPCR groups (Additional file 1: Figure S5).

**Figure 2:**
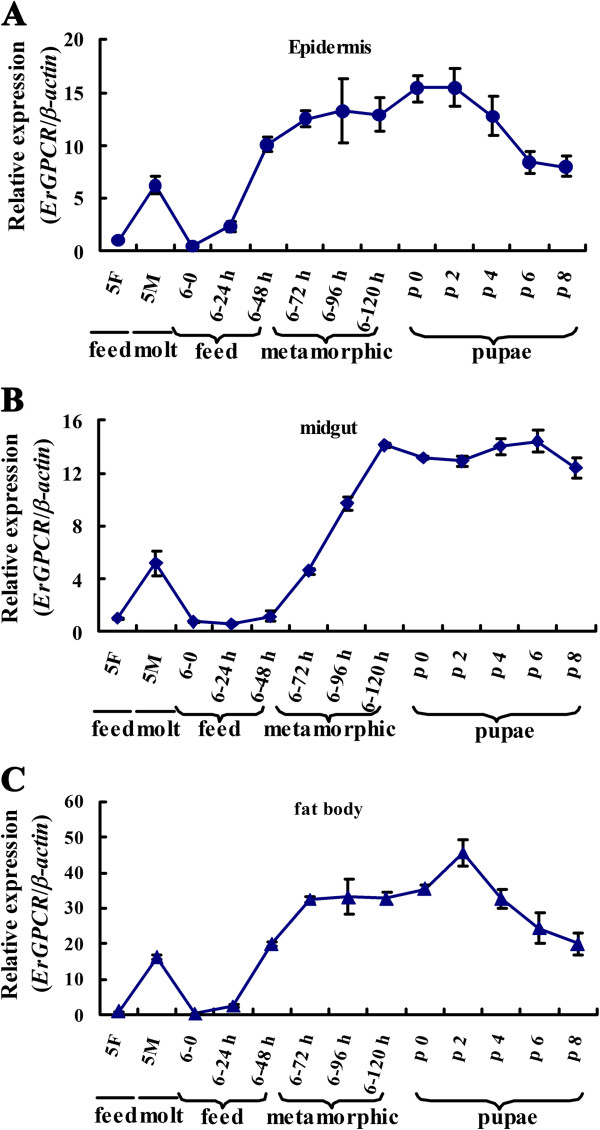
**The increased transcripts of *ErGPCR *during molting and metamorphosis. (A)**, **(B)** and **(C)***ErGPCR* is highly expressed during molting and metamorphosis in epidermis, midgut and fat body detected by qRT-PCR. **5 F** is the fifth instar 12 h larvae; **5 M** is the fifth instar molting larvae; **6**–**0 to 6–120 h** are the 6th instar larvae in hours; p 0 to p 8 are the pupae in days.

**Figure 3:**
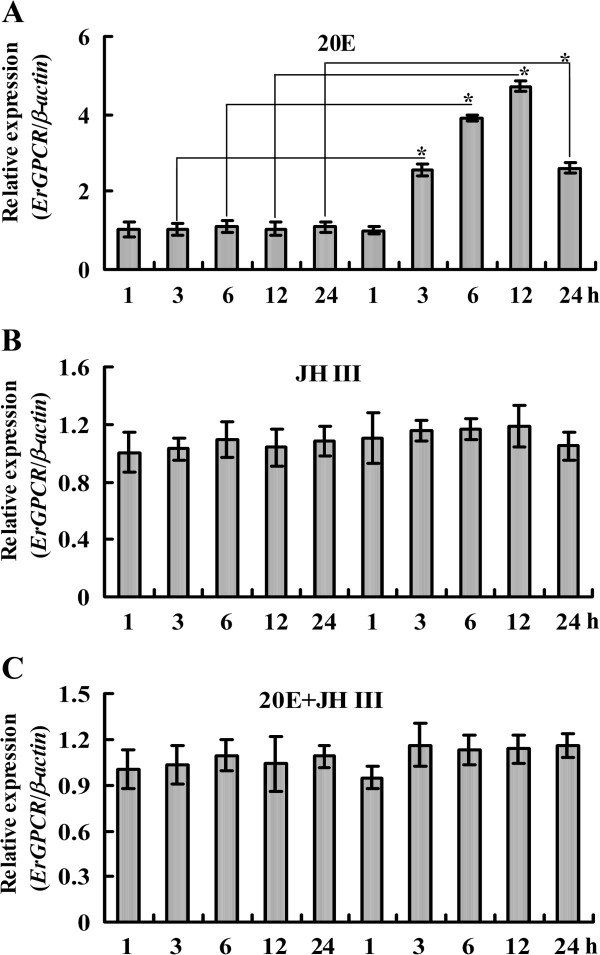
**Hormonal induction of *ErGPCR *in the midgut by injection of 20E (A) or JH III (B) or 20E + JH III (C) into the 6th instar 6 h larvae (500 ng/larva) analyzed by qRT-PCR.** DMSO is a solvent control. *β-actin* gene was used as the quantitative control for the mRNA. The asterisks indicate significant differences between 20E treatment and DMSO solvent control by student’s *t* test analysis from three independent repeats (*p < 0.05, n = 3).

The transcript level of *ErGPCR* was increased at the larval molting stage (5 M) and metamorphic molting stage (sixth-instar 72 h larvae to pupae) in the tissues (Figure 2). Given that the 20E titer is higher during molting and metamorphosis in lepidopteran insect *Manduca sexta*[27], the hormone induction on the mRNA levels of *ErGPCR* was examined. The *ErGPCR* transcript level was upregulated in the midgut from 3 h to 24 h after 20E injection into the sixth-instar larvae. JH III injection into the sixth-instar larvae did not affect the *ErGPCR* transcript levels, but repressed the 20E-induced upregulation of *ErGPCR* (Figure 3). These data suggest that *ErGPCR* mRNA level is upregulated by 20E signaling. To confirm that 20E upregulates *ErGPCR*, we knocked down the nuclear receptor of 20E, *EcRB1*, and analyzed the transcript of *ErGPCR*. When *EcRB1* was knocked down, the upregulation of *ErGPCR* induced by 20E was blocked (Additional file 1: Figure S6). These results reveal that 20E upregulates *ErGPCR* transcript via the nuclear receptor *EcRB1*.

### ErGPCR is involved in the larval–pupal transition *in vivo* by regulating gene expression

**Figure 4:**
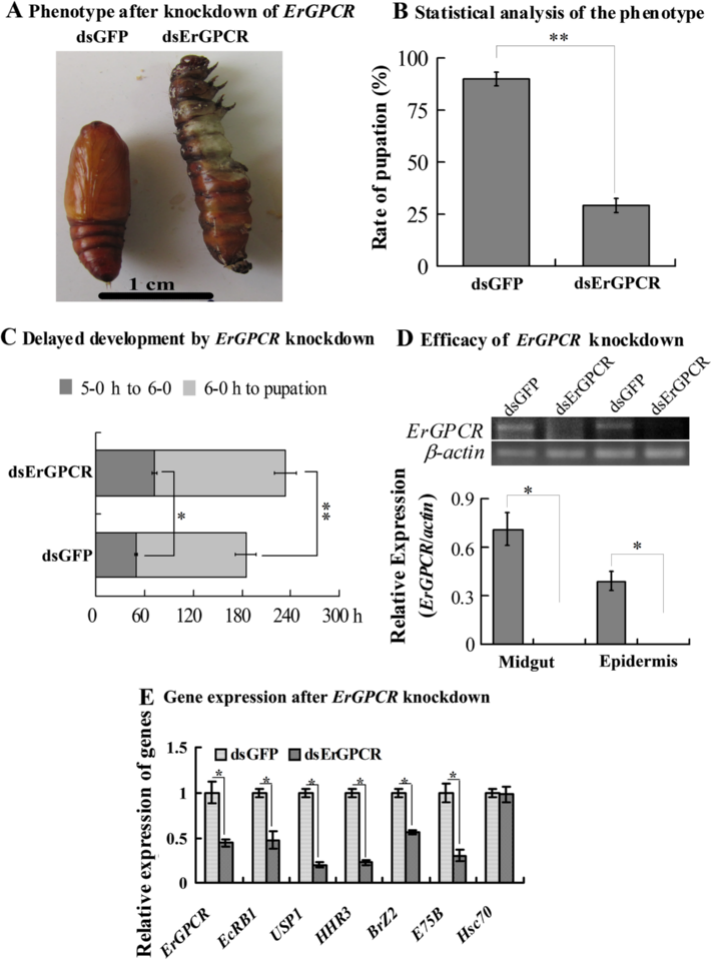
***ErGPCR *knockdown by dsRNA injection blocks larval-pupal transition. (A)** Phenotypes after *ErGPCR* knockdown (*dsErGPCR* injection into the 5th instar larval hemocoel). Scale bar = 1 cm. **(B)** Statistical analysis of the phenotype in **A**. **(C)** 5–0 h to 6–0 h: 0 h of the 5th instar to 0 h of the 6th instar 0 h; 6–0 h to pupation: 0 h of the 6th instar to pupation. **(D)** The efficiency of *ErGPCR* knockdown, analyzed by semi**-**quantitative RT-PCR. **(E)** The gene transcripts in the larval epidermis after *ErGPCR* knockdown were determined by qRT-PCR analysis. Error bars represent the standard deviations of three replicates. Asterisks denote significant differences (*p < 0.05; **p < 0.01, via Student’s *t* test) based on 30 larval samples with three replicates.

The function of *ErGPCR* in larval–pupal transition was determined through RNAi by injecting *dsErGPCR* into the larval hemocoel. The knockdown of *ErGPCR* blocked larval–pupal transition (Figure 4A). In the dsRNA of green fluorescent protein (*dsGFP*)-injected control, 90% of the larvae pupated, whereas 10% died. However, in *dsErGPCR* treatment, only 29% of the larvae pupated, 50% died, and 21% displayed larval–pupal chimeras (Figure 4B). Of the 29% that pupated after *ErGPCR* knockdown, the duration of development was significantly delayed compared with the *dsGFP* control: a 23 h delay from fifth instar to the sixth instar, and a 52 h delay from the sixth instar to the pupal stage (Figure 4C). RT–PCR showed that *ErGPCR* was significantly knocked down by four consecutive *dsErGPCR* injections into the larvae (Figure 4D). The transcript levels of the genes involved in the 20E pathway, including *EcRB1*, *USP1*, *HHR3*, *BrZ2*, and *E75B*, were decreased in the larval epidermis after *ErGPCR* knockdown (Figure 4E). These results suggest that ErGPCR is related to larval–pupal transition and gene expression *in vivo*.

### ErGPCR is located on the membrane and is necessary for 20E-induced gene expression

**Figure 5:**
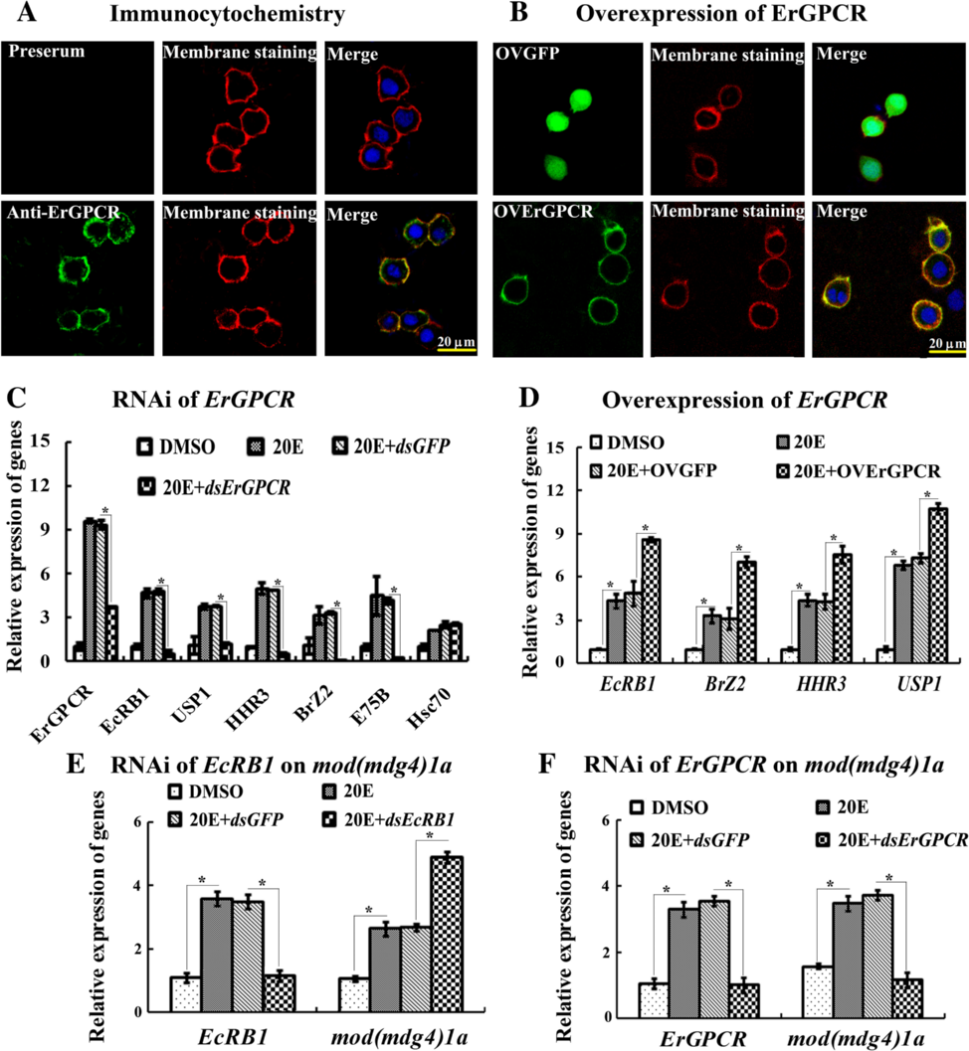
**ErGPCR is located on the plasma membrane and is involved in 20E-induced gene expression in HaEpi cells. (A)** Green fluorescence indicated ErGPCR protein detected with anti-ErGPCR (antibody specificity is shown in Additional file 1: Figure S7) and ALEXA 488–labeled goat anti-rabbit secondary antibody by confocal microscope. The nuclei were stained with 4′-6′-diamidino-2-phenylindole dihydrochloride (DAPI). **(B)** Green fluorescence protein (GFP, green) alone was detected in the entire cell; the plasma membrane (red) was marked with 1,1′-dioctadecyl-3,3,3′,3′-tetramethylindocarbocyanine perchlorate (DiI); and the orange color are the overlapping of ErGPCR-GFP (green) and membrane (red) by confocal microscope. Scale bar = 20 μm. **(C)** and **(D)** The effects of *ErGPCR* knockdown and overexpression on 20E-induced gene expression, analyzed by qRT-PCR. **(E)** and **(F)** 20E upregulated *mod(mdg4)1a* expression not through EcRB1 but through ErGPCR, by qRT-PCR analysis. *β-actin* was used as the quantitative control. * indicates significant differences (*p* < 0.05) among the treatments via Student’s *t* test from three independent replicates.

Immunocytochemical analysis using rabbit anti-ErGPCR polyclonal antibodies showed that ErGPCR was located on the plasma membrane of HaEpi cells. The green fluorescence of ErGPCR overlapped with the red-stained cell membrane to display an orange color by confocal laser scanning microscopy (Figure 5A). Moreover, the overexpression of ErGPCR-GFP (C-terminal with GFP) was also located on the cell plasma membrane (Figure 5B). *ErGPCR* knockdown in the HaEpi cells decreased the 20E-induced transcript levels of *EcRB1*, *USP1*, *HHR3*, *BrZ2*, and *E75B* compared with the *dsGFP*-treated control (Figure 5C). By contrast, ErGPCR overexpression led to an increase in the transcript levels of *EcRB1*, *BrZ2*, *HHR3*, and *USP1* (Figure 5D). Moreover, 20E upregulated the mRNA level of insulator body protein *mod(mdg4)1a* not through *EcRB1* but through *ErGPCR* (Figures 5E and F). These results suggest that ErGPCR may serve as the initiation step of 20E signal amplification on the plasma membrane as nongenomic or pregenomic action before the hierarchical control of 20E-induced genomic action.

### 20E regulates protein nuclear translocation and phosphorylation via ErGPCR

**Figure 6:**
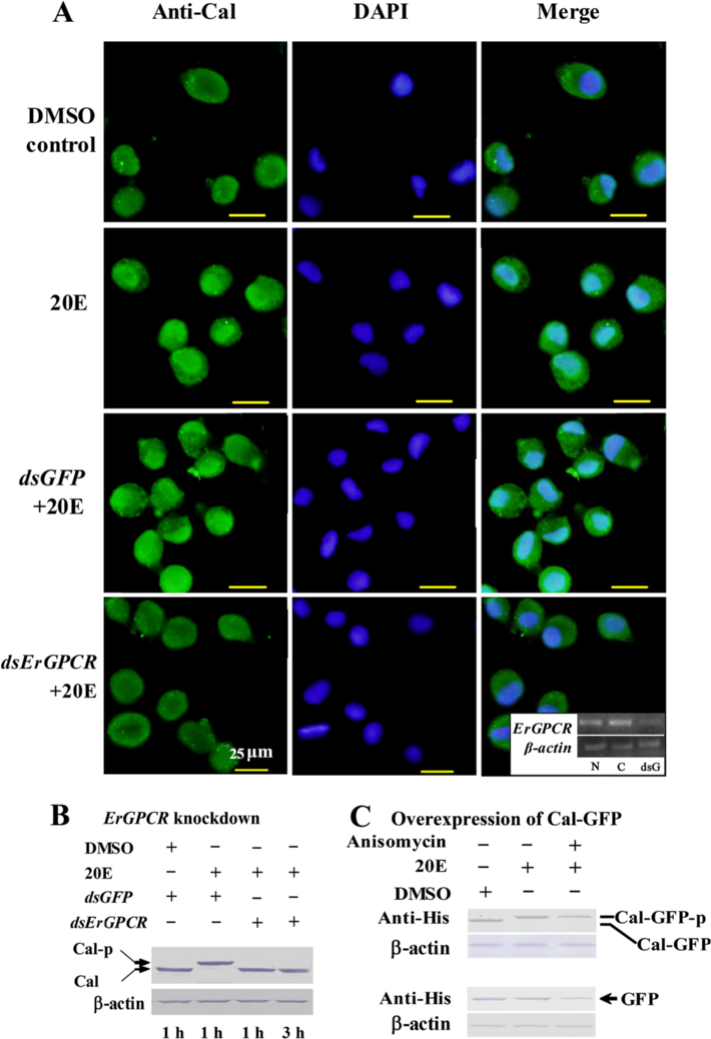
**Knockdown of *ErGPCR *by RNAi blocks 20E-induced nuclear translocation and phosphorylation of Calponin in the HaEpi cells. (A)** Green fluorescence represents Calponin detected with anti-Calponin antibody. Blue fluorescence indicates DAPI-stained nuclei. The image in the white box shows the efficacy of *ErGPCR* knockdown (by dsRNA incubation for 24 h followed by 1 μM 20E induction for 1 h), determined by RT-PCR; N, C, and dsG indicate *ErGPCR* transcripts in normal cells, *dsGFP-*treated cells, and *dsErGPCR-*treated cells, respectively. **(B)** 20E-induced phosphorylation of Calponin was detected with anti-Calponin antibody by western blot analysis*.* Cal-P indicates phosphorylated Calponin. **(C)** The effect of anisomycin on 20E-induced phosphorylation of Calponin was analyzed using anti-His monoclonal antibody by western blot analysis. Cal-GFP (56 kDa) is the fused expressed Calponin with GFP in the HaEpi cells; Cal-GFP-P indicates phosphorylated Calponin-GFP; GFP (35 kDa) was expressed alone in the cells as a control. The cells were incubated with 10 μM anisomycin for 1 h and then treated with DMSO or 1 μM 20E for 1 h.

The nongenomic pathway is characterized by rapid protein translocation and phosphorylation [31]. The Calponin protein has been demonstrated to undergo quick nuclear translocation and phosphorylation by 20E induction [26]. Thus, *ErGPCR* was knocked down in the HaEpi cells to determine its function in 20E-regulated rapid translocation and phosphorylation of Calponin. Calponin was mainly localized in the cytoplasm in the DMSO-negative control cells, but was translocated into the nucleus after 20E induction. However, after *ErGPCR* knockdown, 20E could not induce the nuclear translocation of Calponin (Figure 6A). Moreover, the 20E-mediated Calponin phosphorylation was suppressed when *ErGPCR* was silenced (Figure 6B). The protein synthesis inhibitor anisomycin did not inhibit 20E-induced Calponin phosphorylation (Figure 6C). These results suggest that 20E regulates Calponin nuclear translocation and phosphorylation via ErGPCR.

### 20E regulates cellular Ca^2+^ release and influx via ErGPCR to regulate gene expression

**Figure 7:**
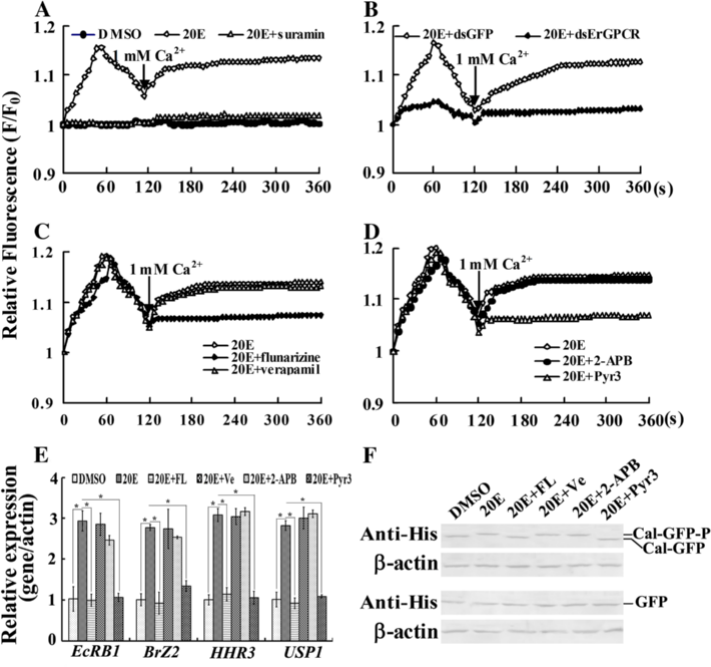
**ErGPCR is involved in the 20E-induced increase in cytosolic Ca**^**2+ **^**levels. (A)** Suramin inhibited the 20E-induced Ca^2+^ increase (1 μM 20E, 1 mM calcium chloride, 50 μM suramin). F represents the fluorescence of cells after treatment, whereas F_0_ denotes the average fluorescence of cells before treatment. Fluorescence was detected every 6 s for 360 s using a Laser Scan Confocal Microscope Carl Zeiss LSM 700 (Thornwood, NY, USA) at 555 nm and then analyzed using the Image Pro-Plus software. **(B)***ErGPCR* knockdown using dsRNA (5 μg/mL) incubation inhibited the 20E-induced Ca^2+^ increase. **(C)** The T-type calcium channel blocker flunarizine dihydrochloride (FL, 50 μM) inhibited the 20E-indued Ca^2+^ influx. The L-type calcium channel blocker verapamil hydrochloride (Ve, 100 μM) did not affect the 20E-induced Ca^2+^ influx. **(D)** The transient receptor potential (TRP) channel blocker pyrazole (Pyr3, 10 μM) inhibited the 20E-induced Ca^2+^ increase. 2-Aminoethoxydiphenyl borate (2-APB) (10 μM to 100 μM) did not affect 20E-induced Ca^2+^ influx. **(E)** qRT-PCR showing the involvement of Ca^2+^ signal in 20E-induced gene expression. Cells were incubated in 1 μM 20E for 6 h after different inhibitors pretreatment for 1 h and the RNA was isolated for qRT-PCR. *β-actin* was used as the quantitative control. *indicates significant differences (*p* < 0.05) among the treatments via Student’s *t* test from three independent replicates. **(F)** Western blot showing the involvement of Ca^2+^ signal in 20E-induced Calponin phosphorylation. pIEx-4-Cal-GFP was overexpressed in HaEpi cells for 48 h. The cells were treated with 1 μM 20E for 1 h after different inhibitors pretreatment for 1 h. The GFP was overexpressed as controls.

The increase in cellular Ca^2+^ is another characteristic of the nongenomic pathway of steroid hormones [32]. Thus, *ErGPCR* was knocked down in the HaEpi cells to determine the function of *ErGPCR* in the rapid 20E-regulated Ca^2+^ increase. When the cells were incubated in calcium-free buffer (DPBS), cytosolic Ca^2+^ level increased rapidly by 20E treatment, and peaked at approximately 50 s, then declined to a lower level at 120 s. Following the addition of 1 mM calcium into DPBS at 120 s, the cytosolic Ca^2+^ levels gradually increased and then remained constant. However, suramin (50 μM) pretreatment for 1 h inhibited the 20E-induced rapid increase in cytosolic Ca^2+^ levels (Figure 7A). When *ErGPCR* was knocked down by RNAi, the 20E-induced Ca^2+^ increase, including intracellular Ca^2+^ release, and extracellular Ca^2+^ influx, was also inhibited compared with the control (Figure 7B). These findings suggest that 20E induces rapid intracellular Ca^2+^ release and extracellular Ca^2+^ influx via ErGPCR.

Various calcium channel blockers, including the T-type voltage-gated calcium channel inhibitor flunarizine dihydrochloride (FL), L-type calcium channel inhibitor verapamil hydrochloride (Ve) [33], transient receptor potential (TRP) calcium channel store-operated channel (SOC) inhibitor 2-aminoethoxydiphenyl borate (2-APB) [34], and receptor-operated (ROC) TRPC3 channel inhibitor pyrazole (Pyr3) [35], were employed to determine the involvement of calcium channels in 20E-induced extracellular Ca^2+^ influx. The 20E-induced Ca^2+^ influx was restrained by 50 μM FL without affecting intracellular Ca^2+^ release. By contrast, 20E-induced Ca^2+^ release and influx were unaffected by 100 μM Ve (Figure 7C). The 2-APB inhibitor (10 μM to 100 μM) had no effect on the 20E-induced Ca^2+^ release and influx. However, 10 μM Pyr3 suppressed the 20E-induced Ca^2+^ influx, but had no effect on intracellular Ca^2+^ release (Figure 7D). These results reveal that T-type calcium channels and TRPC3 channels are involved in 20E-induced Ca^2+^ flux.

To investigate the effect of the cellular Ca^2+^ increase on the 20E-induced gene expression and 20E-induced Calponin phosphorylation, we performed qRT–PCR and western blot. The 20E-induced upregulation of *EcRB1*, *BrZ2*, *HHR3*, and *USP1* was suppressed by FL and Pyr3 (Figure 7E). Meanwhile, the 20E-induced phosphorylation of Calponin was inhibited (Figure 7F). By contrast, Ve and 2-APB inhibitors had no effect on the 20E-induced gene expression and 20E-induced Calponin phosphorylation. These results show that the 20E-induced rapid intracellular Ca^2+^ increase is required for 20E-regulated gene expression and protein phosphorylation.

**Figure 8:**
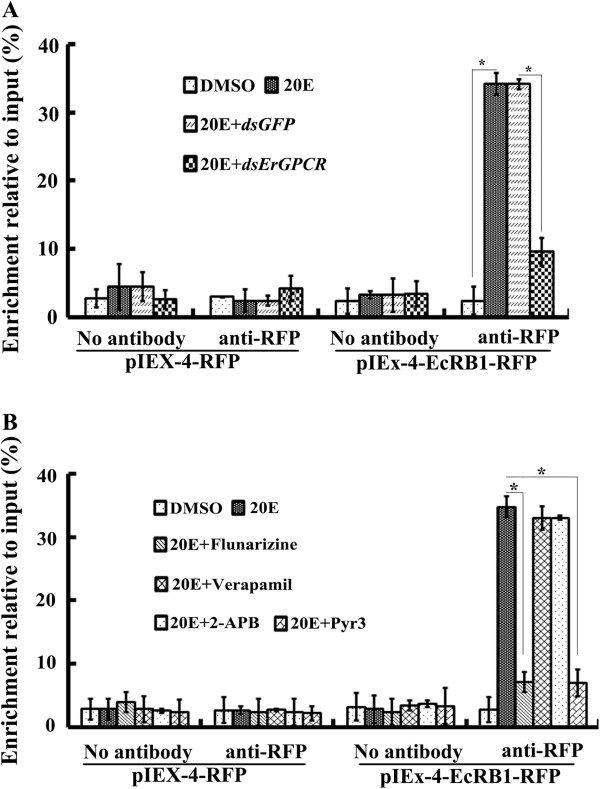
**ChIP analysis showing ErGPCR regulates 20E-induced EcRB1 binding to EcRE through cellular Ca**^**2+ **^**signaling. (A)***ErGPCR* knockdown suppresses the EcRB1 binding to EcRE under 20E treatment. Cells were transfected with pIEx-4-RFP or pIEx-4-EcRB1-RFP for 24 h. The cells were treated by *dsErGPCR* or *dsGFP* for 12 h, then exposed in 1 μM 20E or DMSO for 6 h. **(B)** FL and Pyr3 repress EcRB1 binding to EcRE in 20E treatment. Cells were transfected with pIEx-4-RFP or pIEx-4-EcRB1-RFP for 48 h. The cells were subjected in 1 μM 20E or DMSO for 6 h after different calcium channel inhibitors pretreatment for 1 h. ChIP was carried out without antibody (negative control) or with anti-RFP antibody. The DNA fragment (EcRE) was amplified by qRT-PCR using HHR3F/R primers. Input is the positive control of nonimmunoprecipitated chromatin.

To examine the mechanism by which 20E regulates gene expression through ErGPCR and Ca^2+^ signaling, ChIP experiments were performed by anti-RFP antibody in the EcRB1-RFP-overexpressing HaEpi cells. In *M. sexta*, one ecdysone response element (EcRE1, GGGGTCAATGAACCG) was identified in 20E reporter gene hormone receptor 3 (HR3). 20E regulates EcRB1/USP1 heterodimer binding to EcRE to regulate gene transcription [36]. We cloned the 5' regulatory region of *Helicoverpa* HR3 (HHR3) that contains putative EcRE (GGGGTCAATGAACTG), which has one “T” different from EcRE1 in MHR3. The EcRE from HHR3 is proven to be active by GFP-plasmid examination. Fewer PCR product (EcRE) was detected from the immunoprecipitates in the pIEx-4-RFP-transfected control samples after various treatments by anti-RFP antibody, because the RFP recognized by anti-RFP antibody did not bind to DNA. By contrast, in the pIEx-4-EcRB1-RFP-transfected cells, the PCR product (EcRE) was detected from the immunoprecipitates in 20E induction by anti-RFP antibody. However, after ErGPCR knockdown, the PCR product (EcRE) was significantly decreased compared with the *dsGFP* treatment control (Figure 8A). These results suggest that 20E via ErGPCR regulates EcRB1 binding to EcRE to regulate the 20E-induced gene transcription.

Similarly, in the pIEx-4-RFP-transfected control samples, fewer PCR product (EcRE) was detected in the immunoprecipitate by anti-RFP antibody after various treatments. By contrast, in the pIEx-4-EcRB1-RFP-transfected samples, the PCR product containing EcRE was detected in 20E induction by anti-RFP antibody, which recognized the binding of EcRB1-RFP to EcRE. However, fewer DNA product containing EcRE was detected after the addition of inhibitors FL and Pyr3. Ve and 2-APB had no effect on the PCR product (EcRE) (Figure 8B). These results suggest that 20E via cellular Ca^2+^ signaling modulates the binding of EcRB1 to EcRE, and regulates the 20E-induced gene transcription.

### N-terminal extracellular region is essential to ErGPCR function in the 20E signaling pathway

**Figure 9:**
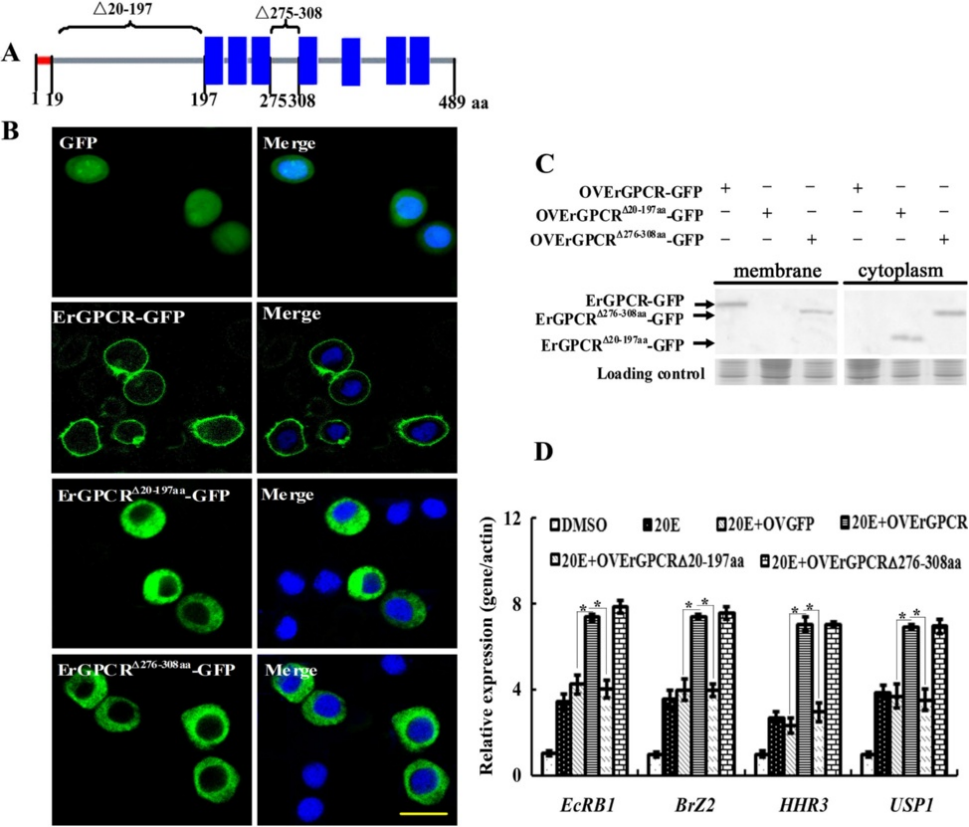
**The N-terminus of ErGPCR is the key domain for its cell membrane localization and function. (A)** mutation sites of ErGPCR, △20-197 indicates the truncation of amino acids 20–197; △275-308 indicates the truncation of amino acids 275–308. **(B)** The localization of the overexpressed GFP (OVGFP), full length of ErGPCR fused with GFP (OVErGPCR-GFP), truncation of N-terminal extracellular region of ErGPCR fused with GFP (OVErGPCR^△20-197aa^-GFP), and truncation of the second inner loop of ErGPCR fused with GFP (OVErGPCR^△276-308aa^-GFP) in the HaEpi cells by confocal microscope. **(C)** Subcellular localization of ErGPCR and two mutants were confirmed by western blot with His-tag antibody. **(D)** The effects of the mutations of ErGPCR on the transcripts of *EcRB1*, *BrZ2*, *HHR3* and *USP1* induced by 20E were examined by qRT-PCR, with *β-actin* as control. Bars indicate mean ± S.D. from three independent experiments. Asterisk indicates significant difference (p < 0.05).

Truncated mutations of ErGPCR in the N-terminal region (aa 20 to 197) (OVErGPCR^∆20–197 aa^) or second inner loop (aa 275 to 308) (OVErGPCR^∆275–308 aa^) were overexpressed by fusing with GFP to analyze the functional domain of ErGPCR in the 20E signaling pathway (Figure 9A). Overexpressed full-length ErGPCR (OVErGPCR) was located on the plasma membrane. However, the N-terminal region-truncated ErGPCR (OVErGPCR^∆20–197 aa^) was in the cytoplasm, whereas the second inner loop truncation (OVErGPCR^∆275–308 aa^) was located in both the plasma membrane and cytoplasm (Figures 9B and C). Correlated with its location, the truncation of the N-terminal extracellular region of ErGPCR (OVErGPCR^∆20–197 aa^) caused a decrease in the 20E-induced transcript levels of *EcRB1*, *BrZ2*, *HHR3*, and *USP1* compared with the full-length OVErGPCR. By contrast, truncation of the second inner loop of ErGPCR (OVErGPCR^∆275–308 aa^) did not affect the 20E-induced transcript levels of *EcRB1*, *BrZ2*, *HHR3*, and *USP1* (Figure 9D). These results indicate that the complete structure of ErGPCR is essential to its plasma membrane location, and the N-terminal extracellular region is required for ErGPCR function in the 20E signaling pathway.

### ErGPCR is not detected to bind with the 20E analog [^3^H]Pon A

**Figure 10:**
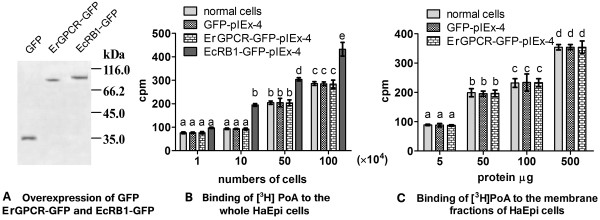
**Binding assay of [**^**3**^**H]Pon A to whole cells or membrane fractions. (A)** Western blot analysis of the overexpressed GFP, ErGPCR-GFP and EcRB1-GFP. **(B)** Binding of [^3^H]Pon A to the whole HaEpi cells that expressed GFP, ErGPCR-GFP or EcRB1-GFP, respectively. The cells (1–100 × 10^4^) were incubated with 0.1 nM [^3^H]Pon A (5740 cpm) for 1 h at 27°C, respectively. **(C)** The membrane fractions (5, 50, 250, 500 μg membrane proteins) isolated from above cells were incubated with 0.1 nM [^3^H]Pon A for 1 h at 27°C, respectively. **cpm:** counts per minute of [^3^H]Pon A. The data marked with different small letters indicate a significant difference at 0.05 levels by the one-way ANOVA with Duncan′s test.

ErGPCR and EcRB1 were overexpressed in HaEpi cells by fusing with GFP at the C-terminus (Figure 10A) for the binding experiments. No increase in [^3^H]Pon A (0.1 nM) was detected with the increase in cell numbers (1 × 10^4^ cells to 100 × 10^4^ cells) in ErGPCR-GFP-transfected cells compared with the normal cells, GFP-overexpressed cells, or EcRB1-GFP-overexpressed cells (Figure 10B). The increase in [^3^H]Pon A was not detected in the plasma membrane fractions (5 μg to 500 μg) from HaEpi cells that overexpressed ErGPCR-GFP compared with that in the membrane fractions from the normal cells and GFP-overexpressed cells (Figure 10C). These results suggest that cells or cell membrane fractions could bind with [^3^H]Pon A in a cell- or cell membrane fraction-dependent manner. However, overexpression of ErGPCR does not increase [^3^H]Pon A binding by this analysis.

## Discussion

Although studies have shown that GPCRs are involved in 20E signaling, definitive evidence of this involvement is scarce. Our study reveals that ErGPCR regulates 20E signaling on the plasma membrane. Through ErGPCR, 20E regulates gene expression, fast protein translocation and phosphorylation, rapid intracellular Ca^2+^ increase, and larval–pupal transition.

### 20E regulates genomic action through the ErGPCR-mediated nongenomic pathway

20E initiates the genomic pathway by binding with its nuclear hormone receptor EcR to regulate gene expression for metamorphosis [37]. 20E upregulated the mRNA levels of *EcRB1*, *USP1*, *HHR3*, *BrZ2*, and *E75B*. Knockdown of ErGPCR repressed the binding of EcRB1 to EcRE thus blocked 20E-induced expression of *EcRB1*, *USP1*, *HHR3*, *BrZ2*, and *E75B* in the cell line and larvae, which resulted in blocking the 20E genomic pathway, thereby inhibiting metamorphosis. These results indicate that 20E initiates a nongenomic pathway to regulate a 20E-mediated genomic pathway via ErGPCR. In positive feedback, ErGPCR transcript was upregulated by 20E via EcRB1. 20E does not act through *EcRB1* to upregulate the mRNA level of the insulator body protein *mod(mdg4)1a* in HaEpi cells [38]. Instead, *mod(mdg4)1a* is upregulated by 20E through *ErGPCR*. These results suggest the existence of various pathways in 20E signaling. The reason that knockdown of *EcRB1* repressed *ErGPCR* but did not repress *mod(mdg4)1a* might because the time difference of mRNA transcription and protein translation of ErGPCR.

Steroid hormones, such as mammalian estrogen and insect ecdysone, are conventionally thought to exert their actions through binding to intracellular receptors because of their small molecules and lipid solubility. However, growing evidence indicates that steroid hormones also exert rapid cell surface-initiated actions by binding to membrane receptors [39], such as the estrogen membrane receptor GPR30 [14]. Rapid protein subcellular translocation and phosphorylation (within minutes) are the outcomes of a nongenomic signaling pathway [24]. 20E regulates the rapid nuclear translocation and phosphorylation of Calponin for gene transactivation in *H. armigera*[26]. We found that 20E regulated Calponin nuclear translocation and subsequent phosphorylation through ErGPCR. This finding suggests that 20E functions in the membrane via a nongenomic pathway to regulate protein translocation and phosphorylation, which may contribute to the activation of transcription factors and formation of transcription complexes.

### ErGPCR is involved in 20E-increased cytosolic Ca^2+^ levels

20E increases the cytosolic Ca^2+^ levels by promoting the release of Ca^2+^ from the intracellular endoplasmic reticulum via an unknown GPCR in silkworm silk glands [23]. 20E also regulates Ca^2+^ influx from extracellular sources via an unknown GPCR that activates calcium channels in murine skeletal muscles [25]. Voltage-gated calcium channels are essential in regulating extracellular Ca^2+^ influx in a wide variety of tissues [40]. T-type Ca^2+^ channels are involved in 20E-induced nuclear and DNA fragmentation in silkworm silk glands [23]. GPCRs serve as chaperones and interact with voltage-gated calcium channels to form complexes [41]. Our data show that 20E regulates rapid intracellular Ca^2+^ release and extracellular Ca^2+^ influx through ErGPCR, and T-type voltage-gated Ca^2+^ channels are involved in Ca^2+^ influx.

In addition, we found that the 20E-induced Ca^2+^ influx was also inhibited by the TRP channel inhibitor Pyr3. This result suggests that TRP channels are also involved in 20E-induced Ca^2+^ influx. TRP channels are non-voltage-gated Ca^2+^ channels involved in Ca^2+^ entry [42]. TRP channels are classified into six subfamilies according to their primary structure and function, including ROC and SOC [43]. GPCRs directly or indirectly modulate several TRP channels [44,45]. TRP channels are associated with steroid hormones in mammals [46]. Rapid calcium release or influx in the cells is the outcome of nongenomic signaling. Calcium is an important secondary messenger that regulates numerous essential physiologic processes, including protein kinase C activation, for further protein phosphorylation [47] and gene transcription. In our study, when the cellular Ca^2+^ was blocked by inhibitors, 20E-induced gene expression and the phosphorylation of Calponin were blocked. These findings confirm the function of calcium on gene expression and protein phosphorylation as the secondary messenger, and reveal that 20E regulates the cellular calcium via ErGPCR to regulate the genomic pathway.

### ErGPCR does not bind with the steroid hormone analog [^3^H]Pon A

In classical GPCR signaling pathways, ligands bind to cell surface transmembrane receptors, such as the β2 ARs, and cause conformational changes in their transmembrane and intracellular domains [48]. Numerous studies have reported the binding of several GPCRs with 20E, such as the binding of DmDopEcR with [^3^H]Pon A [24], or an unknown GPCR in the anterior silk gland of silkworms binding with [^3^H]Pon A [22]. However, we did not detect the binding of ErGPCR with [^3^H]Pon A using the whole cells and cell membrane fractions by overexpressing ErGPCR in HaEpi cells. Thus, ErGPCR is likely transiently activated by 20E without any stable ligand binding.

Based on NCBI Blast analysis (http://blast.ncbi.nlm.nih.gov/Blast.cgi), ErGPCR belongs to methuselah-like proteins in the class B secretin GPCR family, but DmDopEcR shows homology with vertebrate ARs [24]. Identification and phylogenetic analysis using amino acid sequences show that ErGPCR differs from GPR30, beta-2 AR, or *Drosophila* DmDopEcR, which may explain the differences in ligand binding activity. Another possibility is the analytical method, which needs further study in next work. GPR30 has shown negligible binding to estrogen (17β-estradiol) in several studies [49], which may be due to the different analytical methods [17]. A major challenge in the study of steroid hormone nongenomic pathways is the binding assay of GPCR with the steroid hormone [50]. Although ErGPCR did not bind ecdysteroid in our study, this result is of particular importance in many cellular responses to 20E, including 20E-induced mRNA levels, protein subcellular translocation and phosphorylation, and cellular Ca^2+^ increase. Whether other GPCRs can bind with 20E needs further exploration.

A total of 800 GPCRs have been discovered in mammals [51], 1000 in *Caenorhabditis elegans*[52], and 200 in *D. melanogaster*[53]. GPCRs involved in steroid membrane signaling may differ among various organisms. GPCRs form homodimers, heterodimers, oligomers, or complexes in the signalsomes of the membrane [54]. The mechanism underlying GPCRs in the 20E membrane pathway requires further study.

## Conclusions

**Figure 11:**
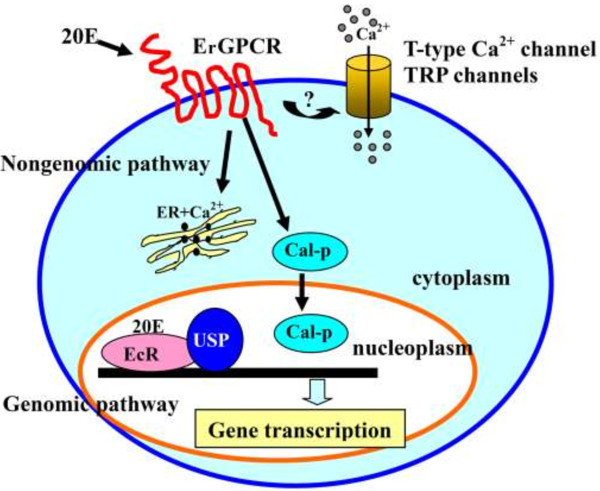
**Diagram showing the ErGPCR-mediated nongenomic pathway response to 20E.** 20E through ErGPCR nongenomic pathway regulates the cytosolic calcium fast increase, including the release of the intracellular Ca^2+^, and the influx of the extracellular Ca^2+^ via T-type calcium channels and TRP channels. 20E also through ErGPCR regulates the rapid phosphorylation and nuclear translocation of Calponin, and through ErGPCR regulates gene transcription in the genomic pathway for metamorphosis.

ErGPCR participates in 20E-regulated gene expression, rapid Calponin nuclear translocation and phosphorylation, rapid intracellular Ca^2+^ release, and extracellular Ca^2+^ influx via T-type calcium channels and TRP channels. The N-terminal extracellular region is critical for the function of ErGPCR in the 20E signaling pathway. ErGPCR is necessary for the larval–pupal transition in *H. armigera* development (Figure 11).

## Methods and materials

### Insect

Cotton boll worms, *H. armigera*, were reared in our laboratory on an artificial diet of wheat, soybean, vitamins, and inorganic salts under a 14 h light:10 h dark cycle at 27°C.

### Quantitative real-time reverse-transcriptase PCR (qRT-PCR)

Approximately 5 μg of the total RNA from each sample was reverse transcribed into first-strand cDNA for qRT-PCR analysis (First Strand cDNA Synthesis Kit, Sangon, China). qRT-PCR was performed using SsoFast™ EvaGreen Supermix (BIO-RAD, Shanghai, China). Thermocycling (94°C for 20 s, 58°C for 20 s, and 72°C for 20 s) was performed for 40 cycles using the CFX96™ real-time system (BIO-RAD). The experiment was repeated three times using three independent RNA samples for statistical analysis. *β-actin* was used as the cDNA quality and quantity controls. The primers used for qRT-PCR are listed in Additional file 1: Table S2.

### Suramin inhibition

*H. armigera* epidermal cells (HaEpi) cells were cultured until 80% confluence at 27°C in 25 cm^2^ culture flasks using Grace’s medium (Invitrogen, Carlsbad, CA, USA) supplemented with 10% fetal bovine serum (FBS, Mdgenics, St. Louis, MO, USA). The cells were incubated with 50 μM suramin (sodium salt; Sigma Chemical, St. Louis, MO, USA) for 1 h and then exposed to 1 μM 20E for another 6 h. Gene expression was determined via qRT-PCR.

### Screening of the target GPCR by qRT-PCR

dsRNA was produced using a MEGAscript™ RNAi kit (Ambion, Austin, TX, USA). Some GPCR ESTs obtained through random sequencing of the HaEpi cells, a cell line previously established in our laboratory, were individually knocked down using RNA interference. Lipofectamine 2000 (Invitrogen, Carlsbad, CA, USA) was used for dsRNA transfection. Briefly, 5 μg of dsRNA and 8 μL of Lipofectamine 2000 were added to 125 μL of FBS-free Grace’s medium incubated at room temperature for 30 min. The reagents were mixed and incubated for another 20 min, and then directly added into HaEpi cells in a six-well plate containing 0.8 mL of Grace’s medium per well. After incubation at 27°C for 24 h, the cells were rinsed and then re-fed with fresh medium containing 10% FBS. The cells were cultured with 1 μM 20E for 6 h. Control cells were treated with the same amount of *dsGFP*. Total RNA was then extracted from the cells for qRT-PCR based on three independent replicates.

### Cloning of full-length cDNA of *ErGPCR*

We obtained an EST with the 3′ end of *ErGPCR* by random sequencing of the cDNA library of the insect during metamorphosis. The 5′ end of the gene was amplified via PCR using the gene-specific reverse primer ErGPCRF1 and the 5′ primer through the Genome Walker method as described by Clontech Laboratories Inc. (cat. no. 638904; Clontech, CA, USA).

### Recombinant expression of ErGPCR in *Escherichia coli* and antiserum preparations

The *ErGPCR* fragment was amplified using the primers ErGPCRExpF and ErGPCRExpR. The PCR product was cloned into pET30a plasmid, expressed in *Escherichia coli* rosette cells, and then cultured in a Luria–Bertani medium (1% tryptone, 0.5% yeast extract, 1% NaCl, and 25 μg/mL kanamycin). The target protein was purified using His-bind resin to produce polyclonal rabbit antiserum. The specificity of the antibody was determined via western blot analysis using horseradish peroxidase–labeled goat anti-rabbit polyclonal secondary antibodies (Zhongshan, Beijing).

### Immunocytochemistry

The cells grown on cover slips were fixed with 4% paraformaldehyde in phosphate-buffered saline (PBS; 140 mM NaCl, 2.7 mM KCl, 10 mM Na_2_HPO_4_, 1.8 mM KH_2_PO_4_, pH 7.4) for 10 min. The fixed cells were incubated with 0.2% Triton-X 100 in PBS for 8 min, blocked with 2% bovine serum albumin (BSA) in PBS for 30 min, and then incubated with primary antibody against the target gene (1:100 dilution in 2% BSA/PBS) overnight at 4°C. The cells were washed and then incubated with the ALEXA 488–labeled goat anti-rabbit secondary antibodies (diluted 1:1000 in 2% BSA/PBS) for 1 h at 37°C. Nuclei were stained with DAPI (1 μg/mL in PBS) for 10 min. Fluorescence was detected using a Laser Scan Confocal Microscope Carl Zeiss LSM 700 (Thornwood, NY, USA).

### ErGPCR overexpression and truncated mutation of ErGPCR

PCR was used to prepare truncated mutations of ErGPCR. ErGPCR fragments were amplified via PCR with various primers (Additional file 1: Table S2) using proofreading DNA polymerase. The mutated ErGPCR was amplified via PCR using the ErGPCR fragments as templates. The open reading frame of *ErGPCR* and different mutated ErGPCRs were inserted into the pIEx-4 plasmid (Merck, Darmstadt, Germany), fused with GFP. The plasmid was transfected into HaEpi cells with Cellfectin following the protocol of the supplier (Invitrogen, Carlsbad, CA, USA). Afterward, 20E was added to the cells at a final concentration of 1 μM. An equal volume of DMSO was used as the solvent control for 20E. DiI (1,1′-dioctadecyl-3,3,3′,3′-tetramethylindocarbocyanine perchlorate; Beyotime, Shanghai, China) was used for plasma membrane staining.

### Examination of Calponin translocation and phosphorylation

Subcellular Calponin translocation and phosphorylation were detected by immunocytochemistry and immunoblotting using rabbit polyclonal antibodies against *Helicoverpa* Calponin. After *ErGPCR* knockdown, the cells were treated with 1 μM 20E for 0.5 h to 3 h. Control cells were treated via the same method using *GFP* dsRNA. Fluorescence was detected using an Olympus BX51 fluorescence microscope. The phosphorylation analysis was performed by western blot.

### Calcium ion detection

HaEpi cells were seeded and cultured for 72 h in a six-well tissue culture plate with 10% FBS Grace’s medium at 27°C. The cells were incubated with dsRNA for 24 h as previously described. The cells were incubated in a 3 μM AM ester Calcium Crimson™ dye (Invitrogen, Carlsbad, CA, USA) in Grace’s medium for 30 min at 27°C. The cells were then washed with DPBS (2.7 mM KCl, 1.5 mM KH_2_PO_4_, and 8 mM Na_2_HPO_4_) and exposed to 1 μM 20E in DPBS for 2 min for detection of intracellular calcium release. Afterward, 1 mM calcium chloride was added to induce extracellular calcium influx. Fluorescence was detected at 555 nm every 6 s for 360 s using a Laser Scan Confocal Microscope Carl Zeiss LSM 700 (Thornwood, NY, USA). Data were analyzed using the Image Pro-Plus software. For the inhibition experiments, the cells were pretreated with different inhibitors for 1 h prior to 20E treatment. The GPCR inhibitor suramin, T-type voltage-gated calcium channel inhibitor flunarizine dihydrochloride, L-type calcium channel inhibitor verapamil hydrochloride, and TRP channel inhibitors 2-APB and Pyr3 were purchased from Sigma Chemical (St. Louis, MO, USA).

### Chromatin immunoprecipitation (ChIP)

The HaEpi cells were seeded in a six-well plate. Cells were transfected with pIEx-4-EcRB1-RFP at a density of 2 × 10^6^. After 24 h, the cells were transfected with *dsErGPCR*, and the controls were incubated with *dsGFP*. After 24 h, the cells were subjected to either DMSO or 1 μM 20E. After 6 h, the cells were cross-linked with 0.5% formaldehyde at 37°C for 10 min, followed by quenching at 0.125 M glycine at room temperature for 10 min. The cells were then washed with ice-cold 1 × PBS and harvested at 6,000 rpm for 5 min. Cells were re-suspended with SDS lysis buffer (1% SDS, 10 mM EDTA, 50 mM Tris–HCl, pH 8.1) and sonicated to yield average DNA fragments of 200 bp to 1000 bp. After centrifugation to remove cell debris, the lysates were pre-cleared with protein A resin at 4°C for 1 h, followed by incubation with no antibody (negative control) or anti-RFP antibody overnight. Immunoprecipitated protein-DNA complexes were incubated with protein A for an additional 2 h at 4°C. The complexes were washed with low-salt buffer [0.1% SDS, 1.0% Triton X-100, 2 mM EDTA, 200 mM Tris–HCl (pH 8.0), 150 mM NaCl] once, high-salt wash buffer [0.1% SDS, 1.0% Triton X-100, 2 mM EDTA, 20 mM Tris–HCl (pH 8.0), 500 mM NaCl] once, LiCl wash buffer [10 mM Tris–HCl (pH 8.1), 0.25 M LiCl, 1 mM EDTA, 1% NP-40, 1% deoxycholate] once, and TE buffer [10 mM Tris–HCl (pH 8.1), 1 mM EDTA] two times. The bound proteins were eluted with elution buffer (1% SDS, 0.1 M NaHCO_3_). DNA-protein crosslinks were reversed at 65°C overnight, followed by RNase and proteinase K treatment. DNA was purified with phenol/chloroform and ethanol precipitation, and analyzed by qRT–PCR using HHR3F/R primers (Additional file 1: Table S2). The negative control cells were transfected with the same volume of pIEx-4-RFP, and the cells received the same treatment as above.

### RNAi in larvae

T7 promoter-containing PCR primers (GPCRRNA-iF, GPCRRNA-iR, GFPRNAiF and GFPRNAiR in Additional file 1: Table S2) were used to amplify the gene fragments. The PCR product purified with phenol–chloroform was used as a template to synthesize dsRNA using the MEGAscript RNAi Kit, as previously described. The dsRNA was diluted in nuclease-free water to 0.4 μg/μL. Afterward, 5 μL was injected into the fifth instar larvae at 6 h and at 30 h, as well as into the sixth instar at 6 h and 30 h. The controls were injected with *dsGFP*. Three independent experiments were performed using 30 larvae each.

### [^3^H]Pon A binding assays

Cell membranes that express ErGPCR, EcRB1 and GFP were prepared from HaEpi cells with the plasmid ErGPCR-GFP-pIEx-4, EcRB1-GFP-pIEx-4, and GFP-pIEx-4. The details are as follows: cells were collected by centrifugation (1700×g, 10 min, 4°C) and then resuspended in 15 mL of HEPES buffer [20 mM HEPES, 6 mM MgCl_2_, 1 mM ethylene diamine tetraacetic acid (EDTA), 1 mM ethylene glycol bis (2-aminoethyl) tetraacetic acid (EGTA), pH 7.4]. After sonication, the homogenate was centrifuged at 1700×g for 10 min. The resulting supernatant was centrifuged at 48000×g for 1 h at 4°C. The pellet was resuspended in HEPES buffer, and the protein concentration was determined via the Bradford method. For the binding assay, a range of membrane fractions were incubated with 1 nM [^3^H]Pon A (Perkin Elmer, MA, USA) at 27°C for 1 h in 200 μL of binding buffer (20 mM HEPES, 100 mM NaCl, 6 mM MgCl_2_, 1 mM EDTA, 1 mM EGTA). For the saturation experiments, reaction mixtures containing 50 μg of the membrane fraction were incubated at 27°C for 1 h in the presence of the appropriate [^3^H]Pon A concentration in the binding buffer. Nonspecific binding was determined in the presence of 1 μM 20E. After incubation, particulate proteins were collected on glass fiber filters. The filters were then added to 5 mL of scintillation fluid. Radioactivity was determined using a SN-6930 liquid scintillation counter (Shanghai Hesuo Rihuan Photoelectric Instrument Co., Ltd., China). The whole cell binding experiments used the same method but without sonication and membrane preparation.

## Abbreviations

20E: 20-hydroxyecdysone; JH: Juvenile hormone; GPCR: G protein-coupled receptor; EcRB1: Ecdysone nuclear receptor B1; USP1: Ultraspiracle protein 1; HR3: Hormone receptor 3; BrZ2: Broad isoform Z2; DMSO: Dimethylsulfoxide; dsRNA: Double-stranded RNA; RNAi: RNA interference; GFP: Green fluorescent protein; RFP: Red fluorescent protein; HaEpi: An epidermal cell line from *Helicoverpa armigera*; FBS: Fetal bovine serum; DAPI: 4′-6-diamidino-2-phenylindole dihydrochloride; BSA: Bovine serum albumin; TRP: Transient receptor potential; SOC: Store-operated channel; ROC: Receptor-operated channel; DiI: 1,1′-dioctadecyl-3,3,3′,3′-tetramethylindocarbocyanine perchlorate; 2-APB: 2-aminoethoxydiphenyl borate; Cpm: Counts per minute of [^3^H] Pon A; qRT-PCR: quantitative reverse transcription polymerase chain reaction; ChIP: Chromatin immunoprecipitation; EcRE: Ecdysone response element.

## Competing interests

The authors declare no conflict of interests.

## Authors’ contributions

Mei-Juan Cai performed the overexpression of ErGPCR and Ca^2+^ detection. Du-Juan Dong constructed the pIEx-4-ErGPCR-GFP plasmid. Yu Wang cloned the gene. Wen Liu overexpressed ErGPCR in cell line. Peng-Cheng Liu examined the phosphorylation and translocation of Calponin. Jin-Xing Wang directed the research. Xiao-Fan Zhao designed the studies and wrote the manuscript. All authors read and approved the final manuscript.

## Supplementary Material

Additional file 1 “The data sets supporting the results of this article are included within the article”.
**Figure S1.** Alignment of the GPCR ESTs obtained by random sequencing the *Helicoverpa* epidermal cell line. **Figure S2.** Screen of the target GPCR involved in 20E-induced gene expression by qRT-PCR. **Figure S3.** Nucleotide and deduced amino acid sequence of ErGPCR. **Figure S4.** Multiple alignments of ErGPCR with other G-protein-coupled receptors from different insects or vertebrates. **Figure S5.** Phylogenetic analysis of ErGPCR. **Figure S6.** 20E upregulates ErGPCR through *EcRB1*. **Figure S7.** The recombinant expression of *ErGPCR* fragments in *E. coli*. **Table S1.** Identification of the GPCRs. **Table S2.** Primers used in dsRNA synthesis and qRT-PCR. Click here for file
