# Supplementary material for: G-protein-coupled receptor participates in 20-hydroxyecdysone signaling on the plasma membrane
Source: Cell Commun Signal. 2014 Feb 10;12:9. doi: 10.1186/1478-811X-12-9 (PMC3937218; doi:10.1186/1478-811X-12-9)
Supplement: Additional file 1 — “The data sets supporting the results of this article are included within the article”. Figure S1. Alignment of the GPCR ESTs obtained by random sequencing the Helicoverpa epidermal cell line. Figure S2. Screen of the target GPCR involved in 20E-induced gene expression by qRT-PCR. Figure S3. Nucleotide and deduced amino acid sequence of ErGPCR. Figure S4. Multiple alignments of ErGPCR with other G-protein-coupled receptors from different insects or vertebrates. Figure S5. Phylogenetic analysis of ErGPCR. Figure S6. 20E upregulates ErGPCR through EcRB1. Figure S7. The recombinant expression of ErGPCR fragments in E. coli. Table S1. Identification of the GPCRs. Table S2. Primers used in dsRNA synthesis and qRT-PCR. [file 1478-811X-12-9-S1.doc]

**Supplementary Materials list**

Figure S1. Alignment of six GPCR ESTs obtained by random sequencing the *Helicoverpa* epidermal cell line.

Figure S2. Screen of the target GPCR involved in 20E-induced gene expression by qRT-PCR.

Figure S3. Nucleotide and deduced amino acid sequence of ErGPCR.

Figure S4. Multiple alignments of ErGPCR with other G-protein-coupled receptors from different insects or vertebrates.

Figure S5. Phylogenetic analysis of ErGPCR.

Figure S6. The recombinant expression of ErGPCR fragments in *E. coli*.

Figure S7. 20E upregulates *ErGPCR* through *EcRB1*.

Table S1. Identification of the GPCRs.

Table S2. Primers used in dsRNA synthesis and qRT-PCR.

**
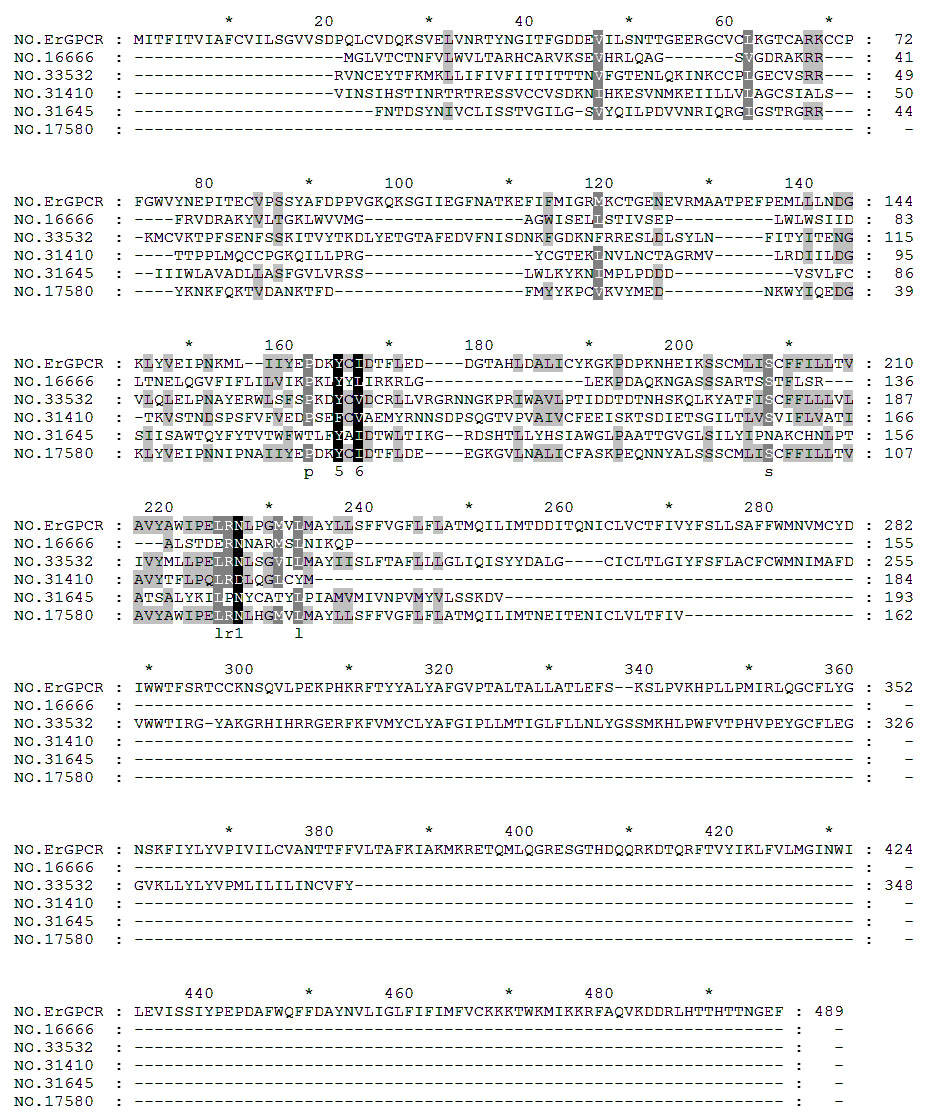
**

**Figure S1. Alignment of the GPCR ESTs obtained by random sequencing the *Helicoverpa* epidermal cell line.**

**
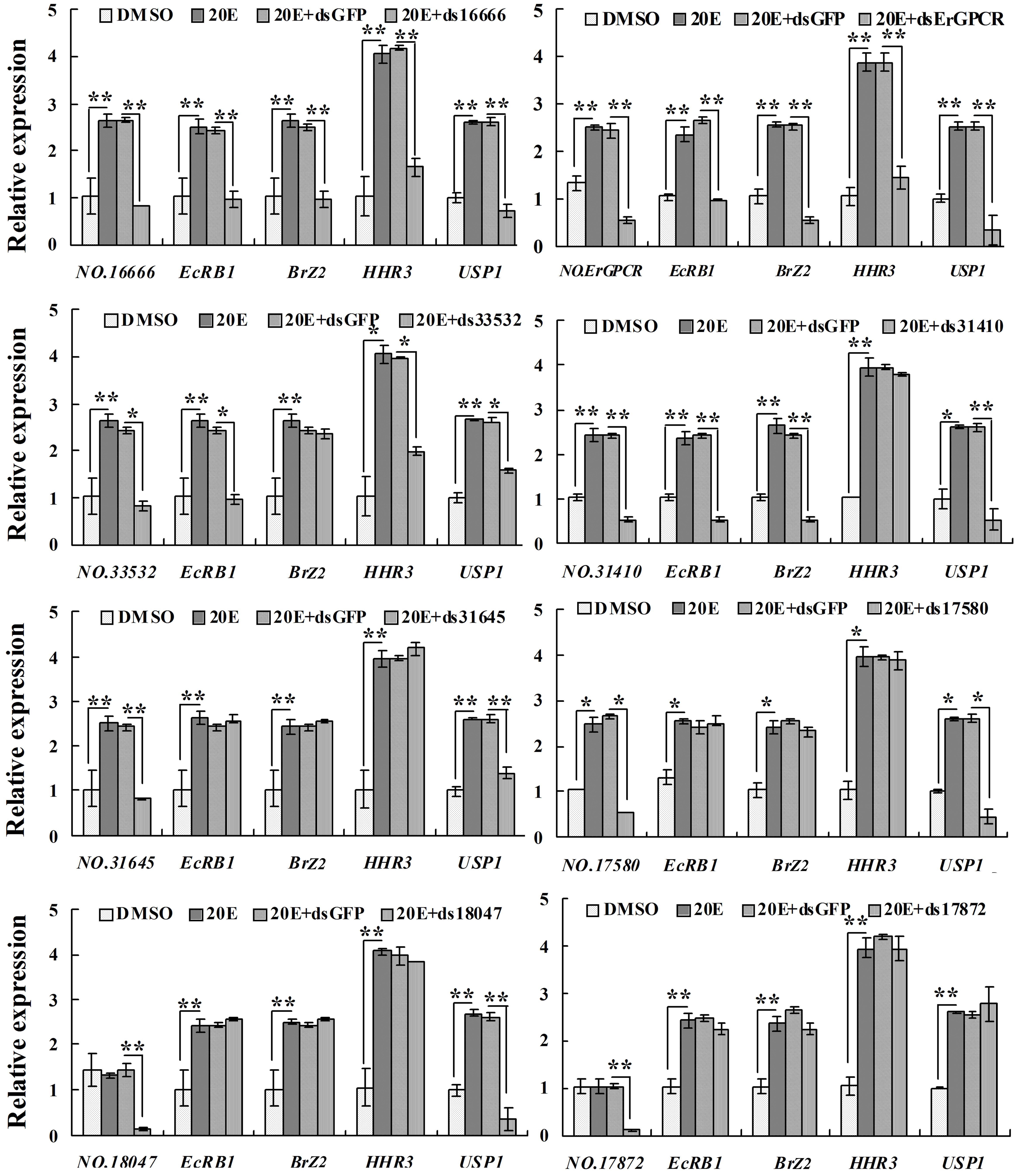
**

**Figure S2. Screen of the target GPCR involved in 20E-induced gene expression by qRT-PCR.** Panels are the expression of *EcRB1*, *BrZ2*, *HHR3* and *USP1* after knockdown of the GPCRs, individually. The primers for synthesis dsGPCRs are in Supplement-Table S2. The cells were incubated with *dsGPCR* at 5 μg/mL for 24 h, then cultured with 20E at 1 μM for 6 h. The experiments were independently repeated three times. The results were based on the ΔΔCT calculation by normalizing of *β-actin* gene. Error bars represent the standard deviation in three independent replicates. Asterisks indicate significant differences (Student’s t test. * p<0.05).

**
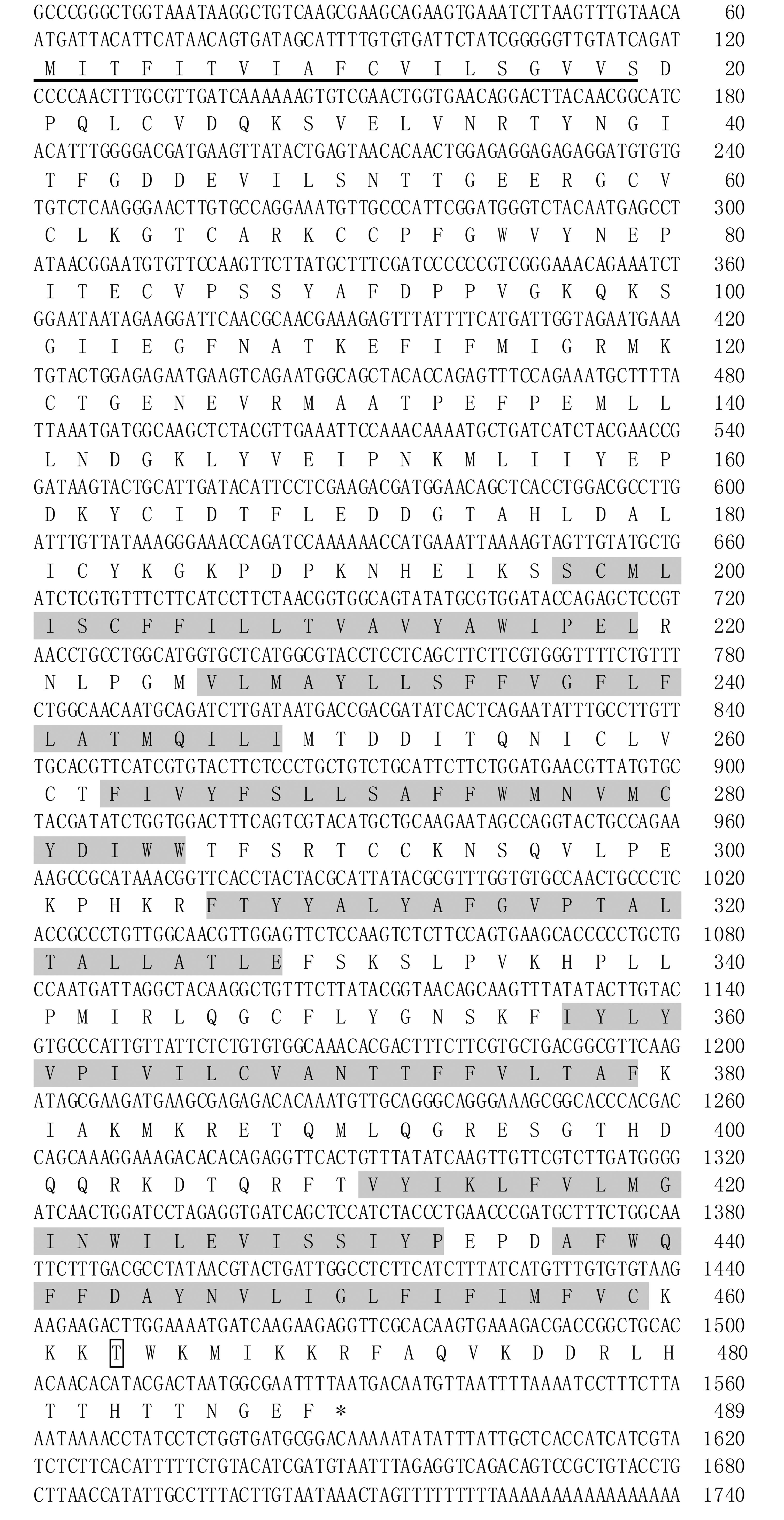
**

**Figure S3. Nucleotide and deduced amino acid sequence of ErGPCR.** The full length of *ErGPCR* cDNA is consisted of 1689 bases with a 1467-base-length open reading frame that encoded a 526-amino-acid-residue protein. The signal peptides (1–19 aa) were underlined. The boxed amino acid (T) was the putative phosphorylation site. The gray background denotes the transmembrane domain (197–219 aa, 226–248 aa, 263–285 aa, 306–328 aa, 357–37 aa, 411–43 aa, 437–459 aa) as determined by analysis (http://smart.embl-heidelberg.de).

**
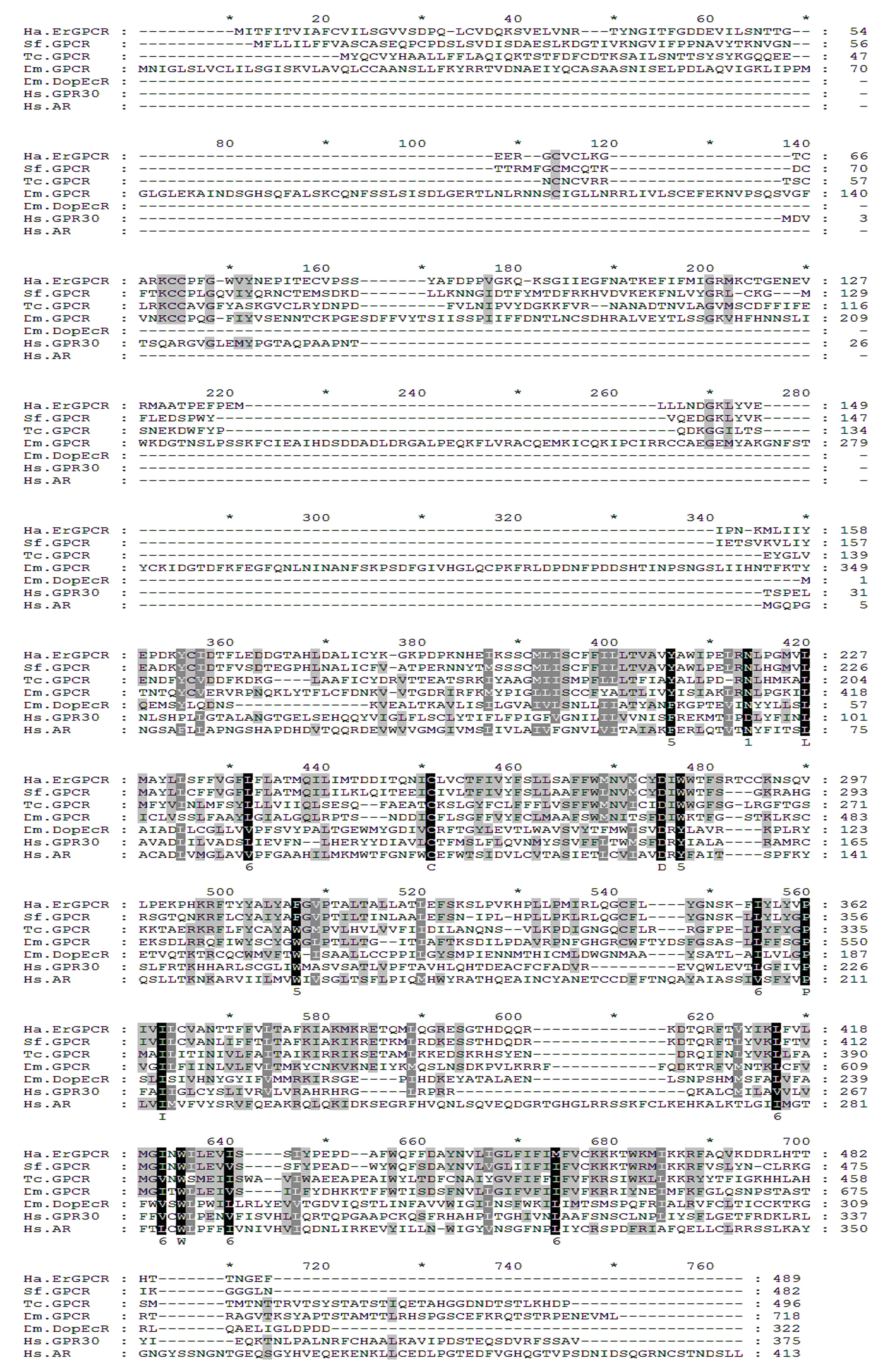
**

**Figure S4.** **Multiple alignments of ErGPCR with other G-protein-coupled receptors from different insects or vertebrates.** *H. armigera* ErGPCR, *S. frugiperda* GPCR (ABC24708.1), *T. castaneum* GPCR (EFA13041.1), *D. melanogaster* (NP_723538.3), *D. melanogaster* DopEcR (NP_647897.2), *H. sapiens* GPR30 (CAG46456.1), and *H. sapiens* AR ( AAF20199.1).

**
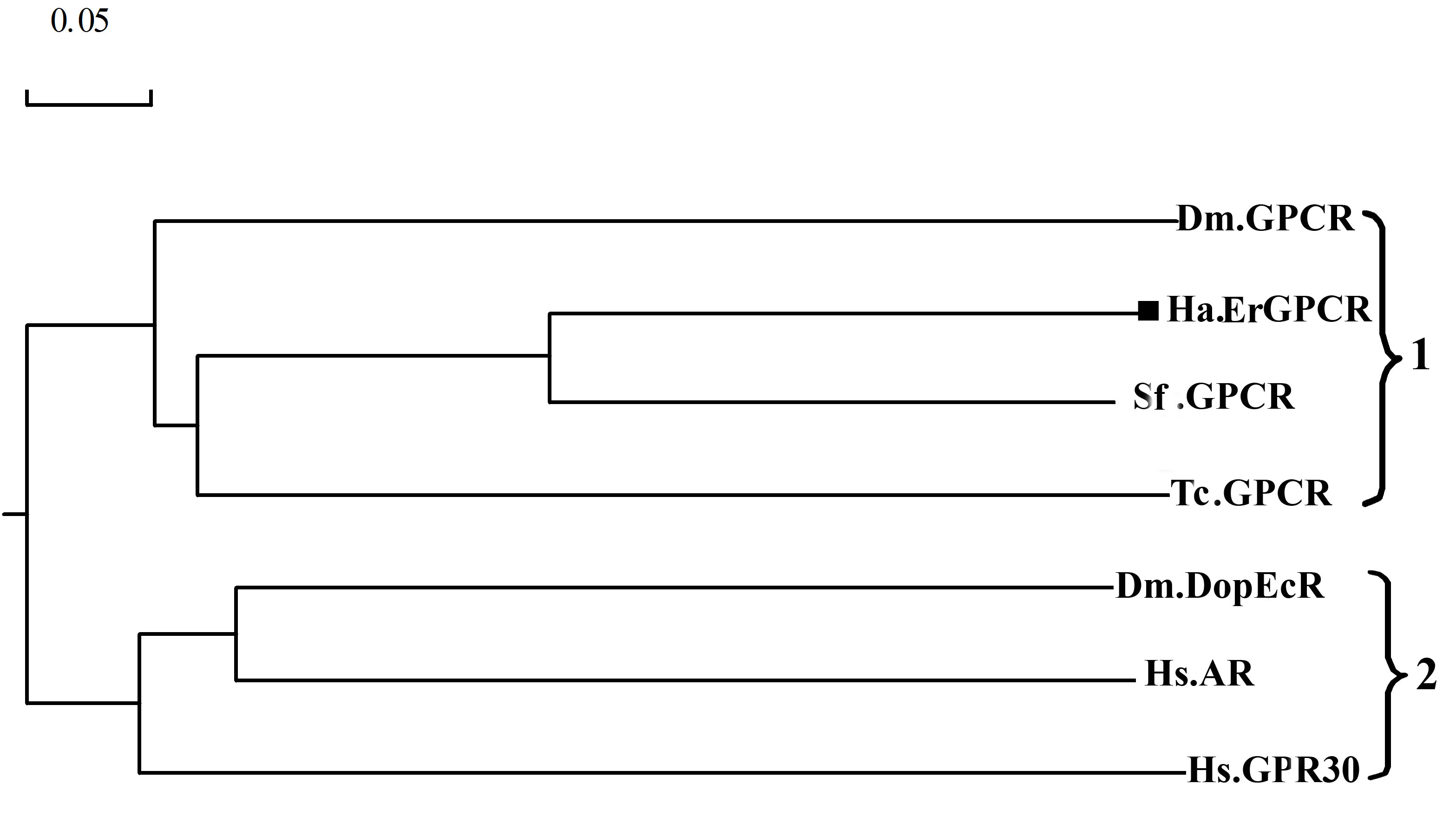
**

**Figure S5. Phylogenetic analysis of ErGPCR.** The sequences used for analysis include: *D. melanogaster* GPCR (NP_723538.3), *H. armigera* ErGPCR, *S. frugiperda* GPCR (ABC24708.1), *T. castaneum* GPCR (EFA13041.1), *D. melanogaster* DopEcR (NP_647897.2), *H. sapiens* GPR30 (CAG46456.1), and *H. sapiens* AR (AAF20199.1).


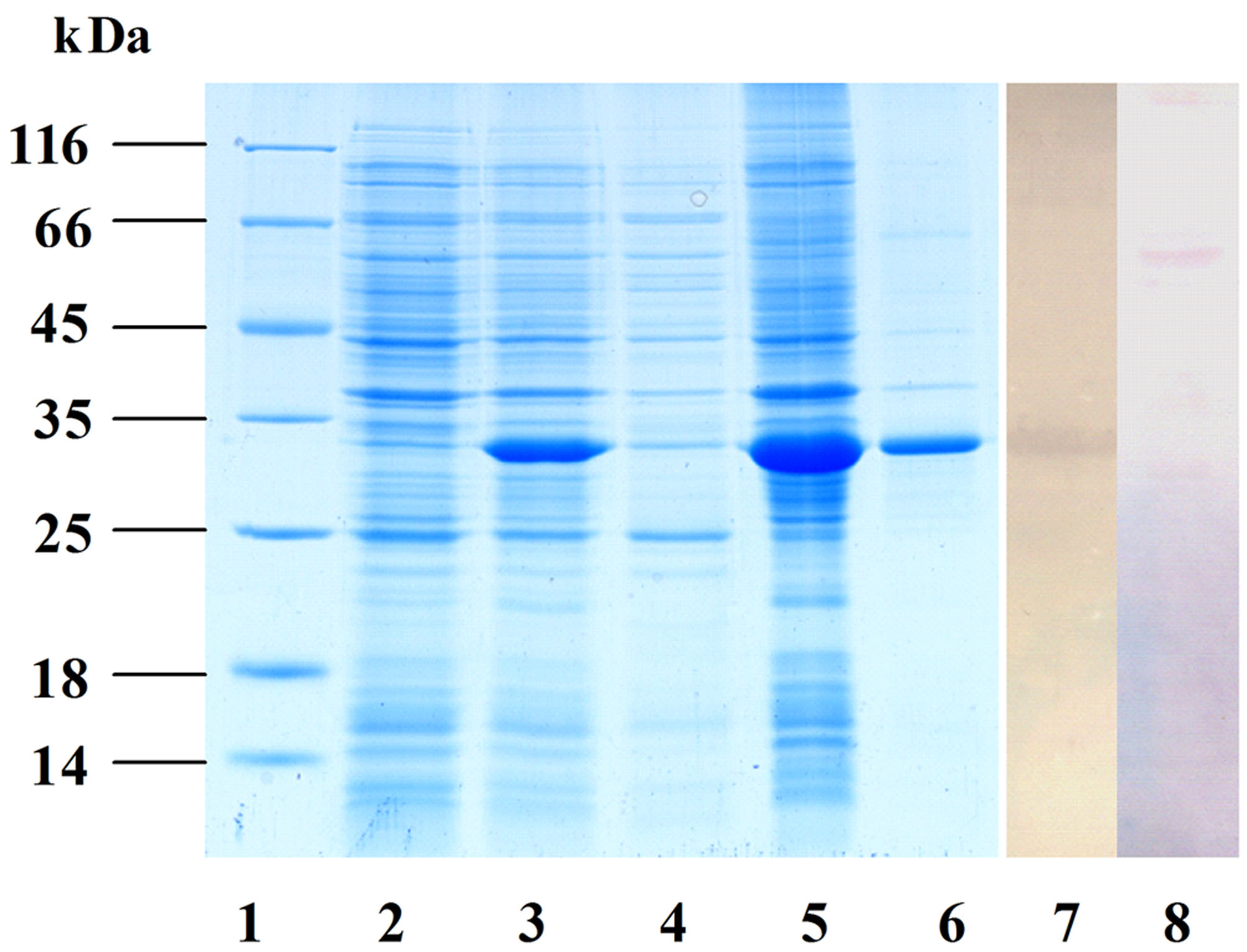


**Figure S6. The recombinant expression of ErGPCR fragment in *E. coli.*** Lanes 1-6, protein marker, total proteins of *E. coli* with ErGPCR–pET30a(+) before induction, induction with IPTG, soluble proteins, insoluble proteins after being sonicated, purified ErGPCR, by SDS-PAGE analysis. Lanes 7 and 8, western blot to determine the specificity of the antibody against the recombinant expressed ErGPCR fragment and the endogenous ErGPCR in the fat body from the 6 d pupae.


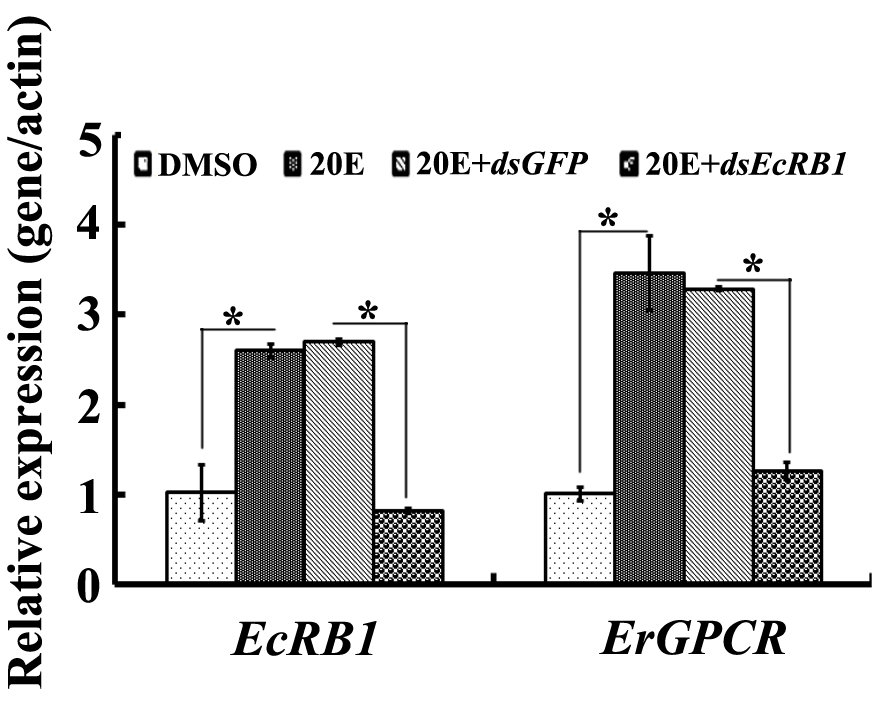


**Figure S7. 20E regulates *ErGPCR* transcript through *EcRB1*.** Cells were transfected with *dsEcRB1*, and the control cells received the same volume of *dsGFP*. After 24 h, cells were subjected to 1 μM 20E for 6 h, and the RNA was isolated for qRT-PCR. The experiments were independently repeated three times. The results were based on the ΔΔCT calculation by normalizing of *β-actin* gene. Error bars represent the standard deviation in three independent replicates. Asterisks indicate significant differences (Student’s t test. * p<0.05).

**Table S1. Identification of the GPCRs**

| **GPCR name** | **Identity analysis *** |
| --- | --- |
| No. 16666  814 bp | [gb|EHJ67961.1](http://www.ncbi.nlm.nih.gov/protein/357612392?report=genbank&log$=protalign&blast_rank=1&RID=59GCE2F4016), hypothetical protein KGM_08449 [*Danaus plexippus*], 74% |
| ErGPCR  1740 bp | [gb|ABC24708.1](http://www.ncbi.nlm.nih.gov/protein/83583697?report=genbank&log$=protalign&blast_rank=1&RID=59GHWK2601R), G protein-coupled receptor [*Spodoptera frugiperda*], 57% |
| No. 33532  1045 bp | [ref|XP_004927377.1,](http://www.ncbi.nlm.nih.gov/protein/512911296?report=genbank&log$=protalign&blast_rank=1&RID=59GRTPXT01R)G-protein coupled receptor Mth-like 3-like [*Bombyx mori*], 44% |
| No. 31410  556 bp | [ref|XP_004924489.1,](http://www.ncbi.nlm.nih.gov/protein/512898814?report=genbank&log$=protalign&blast_rank=1&RID=59H1JTB001R) G-protein coupled receptor Mth-like 2-like [*B. mori*], 58% |
| No. 31645  582 bp | [ref|XP_004930475.1,](http://www.ncbi.nlm.nih.gov/protein/512923938?report=genbank&log$=protalign&blast_rank=1&RID=59HZ56EN01R) G-protein coupled receptor 143-like [*B. mori*], 77% |
| No. 17580  819 bp | [gb|ABC24708.1,](http://www.ncbi.nlm.nih.gov/protein/83583697?report=genbank&log$=protalign&blast_rank=1&RID=59JFXMF7013) G protein-coupled receptor [*S. frugiperda*], 72% |
| No. 18047  1783 bp | [ref|XP_004932194.1,](http://www.ncbi.nlm.nih.gov/protein/512930978?report=genbank&log$=protalign&blast_rank=1&RID=59K7U79Y016) ras guanine nucleotide exchange factor L-like [*B. mori*], 53%, non-7TM |
| No. 17872  1134 bp | [ref|XP_004926858.1](http://www.ncbi.nlm.nih.gov/protein/512909179?report=genbank&log$=protalign&blast_rank=2&RID=59KK5MG501R), uncharacterized protein LOC101737909 [*B. mori*] , 87%, non-7TM |

* The identities were analyzed by the blast search (http://blast.ncbi.nlm.nih.gov/Blast.cgi). The sequences of the GPCR ESTs are in Figure S1.

**Table S2.** Primers used in dsRNA synthesis and qRT-PCR

| Primer names | Primer sequences |
| --- | --- |
| No. 16666-iF | gcgtaatacgactcactataggcgagggtcaagtctgaggtt |
| No. 16666-iR | gcgtaatacgactcactataggttaaggctgtttgatgttga |
| No. 33532-iF | gcgtaatacgactcactataggaatgtgtgagtaggagaaaa |
| No. 33532-iR | gcgtaatacgactcactataggagtagtgggattccaaaggcg |
| ErGPCR-iF | gcgtaatacgactcactatagggttcatccttctaacggtggc |
| ErGPCR-iR | gcgtaatacgactcactatagggtcgcttcatcttcgctatct |
| No. 31645-iF | gcgtaatacgactcactatagg ggacgacgaattattatctg |
| No. 31645-iR | gcgtaatacgactcactatagg cacatctttacttgacaata |
| No. 31410-iF | gcgtaatacgactcactatagg aattaacagcattcattcg |
| No. 31410-iR | gcgtaatacgactcactataggtctgtacatttctgcgacgc |
| No. 18047-iF | gcgtaatacgactcactataggtgatttacctattgccgttga |
| No. 18047-iR | gcgtaatacgactcactataggtgtgtcttgtggtgcttctga |
| No. 17872-iF | gcgtaatacgactcactatagggtctacacccagaaaccgt |
| No. 17872-iR | gcgtaatacgactcactatagggctttgaatagaagctgca |
| No. 17580-iF | gcgtaatacgactcactataggtacaaaaataagtttcaaaag |
| No. 17580-iR | gcgtaatacgactcactatagggcgtagaacacgaagcgtttgt |
| GFPRNAiF | gcgtaatacgactcactataggtggtcccaattctcgtggaac |
| GFPRNAiR | gcgtaatacgactcactataggagctggagacaactcctcacg |
| No. 16666-QF | cgagggtcaagtctgaggtt |
| No. 16666-QR | tattattagtcgtggtggta |
| No. 33532-QF | aggggacgaaacaacggtaaa |
| No. 33532-QR | gcagaagcaagccaggaaaga |
| ErGPCR-QF | aaacggttcacctactacgc |
| ErGPCR-QR | cgcttcatcttcgctatct |
| No. 31645-QF | tcgtaaatcggatacaaagag |
| No. 31645-QR | aagaatagatagtccaacacc |
| No. 31410-QF | tattattagtgctggctgggtg |
| No. 31410-QR | actggtcttggatatctcttcg |
| No. 18047-QF | gctcagaagcaccacaagaca |
| No. 18047-QR | cccgctactacgacaaccatt |
| No. 17872-QF | gggacagaaataagttcacca |
| No. 17872-QR | tccatcacaatcaaaataggg |
| No. 17580-QF | gtcaggaacgcagcacaaac |
| No. 17580-QR | agccacacagagagccacta |
| EcRB1-QF | aattgcccgtcagtacga |
| EcRB1-QR | tgagcttctcattgagga |
| USP1-QF | ggtcctgacagcaatgtt |
| USP1-QR | agctccagctgactgaag |
| BrZ2-QF | ggtgactgtccttactgcggcat |
| BrZ2-QR | ttaattcctttgaccatgact |
| HHR3-QF | tcaagcacctcaacagcagcccta |
| HHR3-QR | gactttgctgatgtcaccctccgc |
| E75B-QF | cgccaactgattctggcat |
| E75B-QR | acaggcatgtcgtcggct |
| Hsc70-QF | gcgtaacaccaccatccc |
| Hsc70-QR | ctgcttctcgtcctcagtcc |
| Actin-QF | agtagccgccctggttgtagac |
| Actin-QR | ttctccatgtcgtcccagt |
| ErGPCRExpF | tactcagaattcagtagttgtatgctgatc |
| ErGPCRExpR | tactcactcgagttacacctctaggatccagtt |
| ErGPCRF1 | gccaccgttagaaggatgaaga |
| 5′-primer | actatagggcacgcgtggt |
| ErGPCROVMNF | tactcactgcagatgattacattcataacagtgatagcattttgtgtaattatatc  ggaggttatatcatataaagggaaacc |
| ErGPCROVMCR | cgtataatgcgtagtaggtgaaccaccagatatcgtagcacataacgttcat |
| ErGPCROVMCF | atgaacgttatgtgctacgatatctggtggttcacctactacgcattatacg |
| ErGPCROVF | tactcactgcagatgattacattcataaca |
| ErGPCROVR | tactcaggtaccaaattcgccattagtcgt |
| HHR3F | tactcagctcttcaactgaacaaaattgcctac |
| HHR3R | agctccaccatggtgaagtcgttagatgccaccac |
